# A chemo-free thermo-sensitive necroptosis-inducing perfusate to enable potent hyperthermic intraperitoneal immunotherapy

**DOI:** 10.1093/nsr/nwag321

**Published:** 2026-05-28

**Authors:** Yujie Zhu, Lin Zhang, Shiqi Yang, Chunjie Wang, Xiangyu Zhang, Ning Li, Zhuang Liu, Liangzhu Feng

**Affiliations:** Institute of Functional Nano & Soft Materials (FUNSOM), Jiangsu Key Laboratory for Carbon-Based Functional Materials & Devices, Biomedical Basic Research Center (BBRC) of Jiangsu Province, Engineering Research Center of RNA Medicine and Cell Therapy Technology, Ministry of Education, Soochow University, Suzhou 215123, China; Department of Obstetrics and Gynecology, The First Affiliated Hospital of Soochow University, Suzhou 215006, China; Institute of Functional Nano & Soft Materials (FUNSOM), Jiangsu Key Laboratory for Carbon-Based Functional Materials & Devices, Biomedical Basic Research Center (BBRC) of Jiangsu Province, Engineering Research Center of RNA Medicine and Cell Therapy Technology, Ministry of Education, Soochow University, Suzhou 215123, China; Institute of Functional Nano & Soft Materials (FUNSOM), Jiangsu Key Laboratory for Carbon-Based Functional Materials & Devices, Biomedical Basic Research Center (BBRC) of Jiangsu Province, Engineering Research Center of RNA Medicine and Cell Therapy Technology, Ministry of Education, Soochow University, Suzhou 215123, China; Institute of Functional Nano & Soft Materials (FUNSOM), Jiangsu Key Laboratory for Carbon-Based Functional Materials & Devices, Biomedical Basic Research Center (BBRC) of Jiangsu Province, Engineering Research Center of RNA Medicine and Cell Therapy Technology, Ministry of Education, Soochow University, Suzhou 215123, China; Department of Radiation Oncology, National Cancer Center/National Clinical Research Center for Cancer/Cancer Hospital, Chinese Academy of Medical Sciences, Peking Union Medical College, Beijing 10021, China; Institute of Functional Nano & Soft Materials (FUNSOM), Jiangsu Key Laboratory for Carbon-Based Functional Materials & Devices, Biomedical Basic Research Center (BBRC) of Jiangsu Province, Engineering Research Center of RNA Medicine and Cell Therapy Technology, Ministry of Education, Soochow University, Suzhou 215123, China; Institute of Functional Nano & Soft Materials (FUNSOM), Jiangsu Key Laboratory for Carbon-Based Functional Materials & Devices, Biomedical Basic Research Center (BBRC) of Jiangsu Province, Engineering Research Center of RNA Medicine and Cell Therapy Technology, Ministry of Education, Soochow University, Suzhou 215123, China

**Keywords:** thermosensitization, necroptosis, thermo-sensitive necroptosis-inducing perfusate, hyperthermic intraperitoneal immunotherapy

## Abstract

Hyperthermic intraperitoneal chemotherapy (HIPEC) is widely performed for treating peritoneal malignancies, yet its clinical application remains limited by insufficient efficacies and risks in chemotherapy-associated side effects. This study demonstrates that mild hyperthermia enhances cancer cell sensitivity to hydrogen peroxide (H_2_O_2_) and Ca^2+^ exposure while sparing normal cells at both elevated and physiological temperatures. Mechanistically, mild hyperthermia promotes H_2_O_2_ cellular entry, synergistically activating calcium channels in the plasma membrane and endoplasmic reticulum to induce Ca^2+^-overload-dependent cancer cell necroptosis. Using H_2_O_2_ and Ca^2+^ solution as a thermo-sensitive necroptosis-inducing perfusate (TNIP), peritoneal perfusion at 43°C demonstrates stronger suppressive effects on the growth of multiple peritoneal tumors in mice compared to conventional HIPEC using various chemotherapeutics. TNIP-mediated hyperthermic intraperitoneal treatment also elicits robust antitumor immunity in syngeneic murine models, with enhanced therapeutic efficacy when combined with postoperative immune checkpoint blockade therapy. The superior immune activation capacities of this strategy are further validated in patient-derived organoids. This work establishes a chemo-free, thermo-activated immunogenic perfusion strategy to selectively trigger cancer cell necroptosis, demonstrating a translatable hyperthermic intraperitoneal immunotherapy with improved therapeutic efficacy and safety.

## Teaser

Mild heat plus simple hydrogen peroxide/Ca^2+^ perfusate can selectively kill cancer cells, boost antitumor immunity, and may offer a safer, chemo-free alternative to standard hyperthermic intraperitoneal chemotherapy.

## INTRODUCTION

Peritoneal malignancies are defined as the primary occurrence of malignant tumor in the peritoneum or secondary progression from cancers of the digestive system or ovaries [1–3]. Hyperthermic intraperitoneal chemotherapy (HIPEC), a procedure involving direct infusion of heated chemotherapeutic drug solution (~43°C) into the abdominal cavity, has been approved as an effective adjuvant or neoadjuvant strategy to surgery for synergistic suppression of peritoneal malignancies [4,5]. Compared with conventional systemic chemotherapy, HIPEC demonstrates advantages in achieving high local drug concentrations in tumor lesion while minimizing systemic exposure, attributed to the peritoneal-plasma barrier [4,6]. Although mild hyperthermia potentiates the efficacy of most chemotherapeutic drugs [7,8], HIPEC would still lead to residual chemotherapeutics-induced side effects, including bone marrow suppression, renal dysfunction, and intraperitoneal infection [9,10]. Moreover, as a local treatment method, HIPEC using existing chemotherapy drugs without potent abilities in activating antitumor immunity, is not able to achieve systemic tumor suppression. Therefore, for patients with advanced cancers, HIPEC is largely utilized as an adjuvant treatment for cytoreductive surgery or a palliative care to delay tumor progression, though both approaches still face challenges due to high rates of tumor relapse.

Accumulating evidence demonstrates that dying cancer cells undergoing various programmed cell death (PCD) modes exhibit distinct immunogenicity in priming tumor-specific immune responses [11–13]. While chemotherapy and radiotherapy enhance the immunogenicity of apoptotic cancer cells by promoting mitochondrial DNA release, their immunostimulatory potency remains limited due to their inherent immunosuppressive nature [14]. In contrast, necroptosis, a well-characterized lytic PCD mediated by receptor-interacting protein kinase-3 (RIPK3) and its down-streaming mixed lineage kinase like (MLKL), demonstrates superior efficacy in initiating antitumor immune responses [15,16]. Unlike apoptosis, whose immunogenicity is strongly suppressed by caspase-dependent processes, necroptosis is a caspase-independent and releases immunostimulatory signals such as damage-associated molecular patterns and proinflammatory chemokines and cytokines [17]. Consequently, inducing necroptosis within the tumor microenvironment represents a viable strategy to initiate robust antitumor immunity via enhancing dendritic cell (DC) mobilization and tumor-specific CD8^+^ T-cell cross-priming [18,19]. Thus, since mild hyperthermia demonstrates potential as an external stimulus for selective tumor eradication [8], developing agents that can selectively induce necroptotic cancer cell death under mild hyperthermia may offer a compelling approach to enable hyperthermic intraperitoneal immunotherapy.

Intracellular Ca^2+^, an important second messenger, plays a crucial role in determining the fate of cancer cells and other cell types [20–22]. Cellular Ca^2+^ overload has been shown to contribute to diverse PCD modes, including necroptosis, via inducing endoplasmic reticulum stress (ERS) and mitochondrial dysfunction, both of which are key intracellular Ca^2+^ storage sites. Mitochondrial Ca^2+^ influx has been demonstrated to trigger excessive reactive oxygen species (ROS) generation through the activation of mitochondrial metabolism [23,24]. Conversely, ROS such as hydrogen peroxide (H_2_O_2_), which can be endogenously generated by nicotinamide adenine dinucleotide phosphate hydrogen (NADPH) oxidase, also act as critical signaling molecules in cell survival and death decision through mutual interactions with Ca^2+^ signaling networks [25,26]. Recently, intracellular Ca^2+^ has been studied as potential therapeutic targets for new cancer treatment development, demonstrating crucial roles in inducing immunogenic PCDs in cancer cells [27–29]. In addition, due to the inherent tolerance of normal cells to oxidative stress, strategies that induce intracellular ROS amplification have been extensively explored to enable selective eradication of cancer cells through different PCDs [30,31].

In this study, we serendipitously discovered that mild hyperthermia (43°C) significantly enhanced the sensitivity of various types of cancer cells to concurrent Ca^2+^/H_2_O_2_ exposure, thereby synergistically inducing lytic cancer cell death with minimal impact on normal cells. Transcriptome sequencing and mechanistic studies showed that mild hyperthermia markedly enhanced cellular H_2_O_2_ uptake, leading to intracellular Ca^2+^ overload through concurrent activation of extracellular Ca^2+^ influx and ER Ca^2+^ release via diverse calcium channels. The Ca^2+^ overload subsequently induced ERS and mitochondrial dysfunction, which in turn promoted intracellular oxidative stress amplification, thereby triggering necroptosis via activating the p-RIPK3-p-MLKL signaling axis. When heated to 43°C and administrated via intraperitoneal perfusion in mice with peritoneal malignancies, the Ca^2+^/H_2_O_2_ solution, designated as thermo-sensitive necroptosis-inducing perfusate (TNIP), demonstrated superior tumor growth suppression compared to conventional 5-fluorouracil (5-Fu) and cisplatin mediated HIPEC treatments. Due to its dual activation of innate and adaptive antitumor immunity, TNIP-mediated hyperthermic intraperitoneal perfusion (HIP) therapy synergized effectively with postoperative immune checkpoint blockade (ICB) immunotherapy, achieving enhanced tumor control in multiple murine tumor models. The superior capacity of this treatment in killing cancer cells and activating antitumor immunity was further validated in patient-derived organoids (PDOs). This work thus presents a chemo-free perfusate with thermo-sensitive necroptosis inducing capacities for hyperthermic intraperitoneal immunotherapy (Fig. S1).

## RESULTS

### Mild hyperthermia synergizes with Ca^2+^/H_2_O_2_ exposure to induce lytic cancer cell death

**Figure 1.**
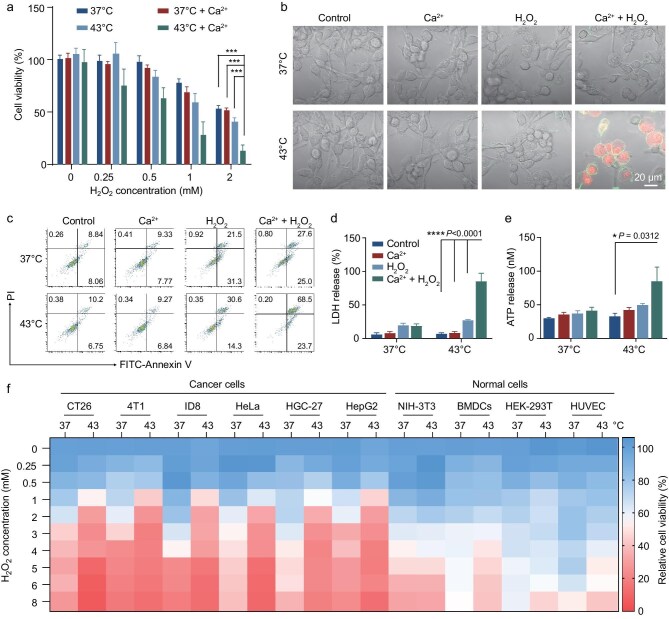
Ca^2+^/H_2_O_2_ co-incubation sensitizes cancer cells to subtherapeutic hyperthermia. (a) Cell viability of CT26 cells under different concentrations of H_2_O_2_ with/without Ca^2+^ (8 mM) exposure and subtherapeutic hyperthermia. (b and c) Confocal images (b) and flow cytometry analysis (c) of CT26 cells stained by FITC-annexin V (green) and PI (red) after various treatments. (d and e) LDH (d) and ATP (e) release from CT26 cells treated with Ca^2+^ and H_2_O_2_ incubation under 37°C or 43°C. (f) Heat map of the cell viability of indicated cells incubated with Ca^2+^ (8 mM) and H_2_O_2_ (varying concentrations as indicated) at 37°C or 43°C. Data are presented as the mean ± standard deviation (SD). Statistical analysis was performed using one-way analysis of variance. (****P < 0.0001, ***P < 0.001, **P < 0.01, and *P < 0.05).

Mild hyperthermia has demonstrated high potency in promoting cellular uptake of diverse substances ranging from ions (e.g., Ca^2+^), molecules, and even nanoparticles via distinct mechanisms [32–34]. Motivated by the synergy between intracellular Ca^2+^ overload and oxidative stress in triggering PCD of cancer cells, we hypothesized that mild hyperthermia might directly enhance the cytotoxicity of extracellular Ca^2+^/H_2_O_2_ exposure. To verify this hypothesis, cell viability assay results first demonstrated that concurrent treatment with Ca^2+^ (8 mM) and H_2_O_2_ at 43°C for 30 min produced marked cytotoxicity in a H_2_O_2_-concentration-dependent manner (Fig. 1a). In contrast, parallel incubations with Ca^2+^ (8 mM) and H_2_O_2_ at 37°C, or H_2_O_2_ alone at both temperatures, showed minimal cytotoxicity. Notably, Ca^2+^ concentrations ≤4 mM failed to significantly enhance the cytotoxicity of H_2_O_2_ (2 mM) under a 30-min 43°C incubation (Fig. S2). A 30-min exposure at 41°C did not substantially enhance the cytotoxicity of Ca^2+^ (8 mM)/H_2_O_2_ (2 mM) co-incubation, whereas a 30-min exposure at 45°C alone caused obvious cytotoxicity (Fig. S3). Collectively, these results indicate that short-term 43°C exposure markedly sensitizes CT26 cells to extracellular Ca^2+^/H_2_O_2_ exposure, and this condition was selected for subsequent experiments.

Next, confocal microscopy was used to evaluate CT26 cell morphology post-treatment. Cells co-treated with Ca^2+^ (8 mM) and H_2_O_2_ (2 mM) at 43°C for 30 min exhibited pronounced plasma membrane blebbing, a hallmark of lytic cell death (Fig. 1b and Fig. S4). In contrast, no blebbing was observed in cells treated with co-incubation of Ca^2+^ (8 mM) and H_2_O_2_ (2 mM) at 37°C or either agent alone at either temperature. Furthermore, only the Ca^2+^/H_2_O_2_ co-incubation at 43°C significantly increased annexin V and propidium iodide (PI) dual positive cells, and triggered lactate dehydrogenase (LDH) and adenosine triphosphate (ATP) release (Fig. 1c–e and Fig. S5).

We investigated the selective cytotoxicity of Ca^2+^/H_2_O_2_ co-incubation across multiple tumor and normal cell lines under varying temperatures and H_2_O_2_ concentrations. Cell viability assays revealed that the Ca^2+^/H_2_O_2_ co-incubation ([Ca^2+^] = 8 mM) at 43°C exhibited significantly stronger cytotoxicity toward both murine cancer cells (e.g., CT26, 4T1, and ID8) and human cancer cells (e.g., HeLa, HGC-27, and HepG2) compared to the treatment at 37°C, in a H_2_O_2_-concentration-dependent manner (Fig. 1f and Fig. S6). In sharp contrast, normal cells including murine bone marrow-derived dendritic cells (BMDCs), murine NIH-3T3 cells, human HEK-293T cells, and human HUVEC cells demonstrated greater tolerance to Ca^2+^/H_2_O_2_ co-incubation at 43°C, with half maximal inhibitory concentration (IC_50_) values ranging from 4.42 to 7.44 mM H_2_O_2_. These values were substantially higher than IC_50_ values (≤1.50 mM) observed for cancer cells. Notably, all cancer cells treated with Ca^2+^ (8 mM)/H_2_O_2_ (2 mM) at 43°C for 30 min showed significant plasma membrane blebbing, whereas no obvious plasma membrane blebbing was observed in normal cells under identical conditions (Fig. S7). These findings demonstrate that Ca^2+^/H_2_O_2_ co-incubation ([Ca^2+^] = 8 mM, [H_2_O_2_] = 2 mM) could synergize with mild hyperthermia (43°C) to selectively induce lytic cell death in diverse cancer cell types while sparing normal cells; at physiological body temperature, the mixture exhibits great biocompatibility. The selective cytotoxicity of Ca^2+^/H_2_O_2_ co-incubation under mild hyperthermia should be ascribed to that cancer cells are more sensitive to both hyperthermia and ROS exposure than normal cells according to previous reports [30,31,35].

### Mild hyperthermia synergizes with Ca^2+^/H_2_O_2_ to induce necroptosis in cancer cells

**Figure 2.**
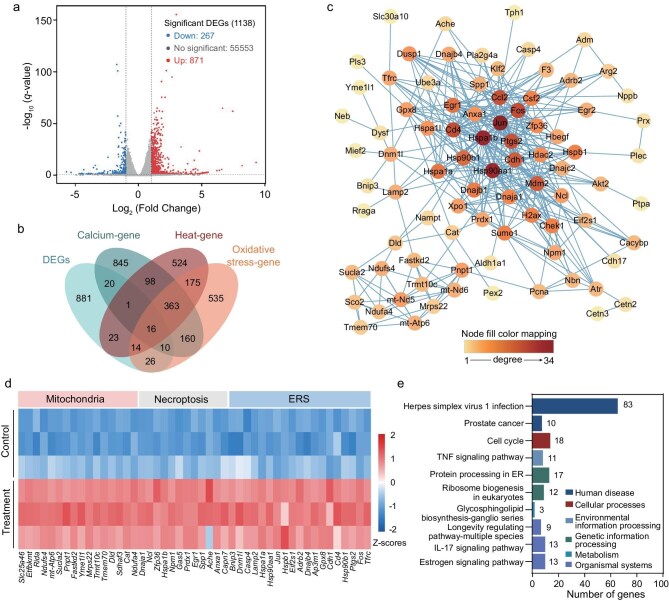
Bulk transcriptome sequencing analysis of cancer cells with Ca^2+^/H_2_O_2_ co-incubation under subtherapeutic hyperthermia. (a) A volcano plot showing the overall distribution of regulated genes. The abscissa denotes the fold change in gene expression across different samples, and the ordinate represents the statistical significance (*q*-value) of gene expression differences. Red, blue, and gray dots signify upregulated, downregulated, and non-significantly changed genes, respectively. (b) Venn diagram of DEGs intersecting with heat, calcium, and oxidative stress-related gene sets. (c) The PPI networks of 110 intersection genes in panel (b), constructed based on the STRING database using Cytoscape software. (d) Heat map of 57 out of 110 intersection genes that overlapped with ERS-related, mitochondrial, and necroptosis-related gene sets. (e) KEGG enrichment analysis of DEGs between the control group and the Ca^2+^ and H_2_O_2_ under 43°C co-incubation group (*n* = 3).

We performed bulk transcriptome analysis to investigate the molecular mechanisms underlying the synergistic effect of Ca^2+^/H_2_O_2_ co-incubation and mild hyperthermia (43°C, 30 min) in inducing lytic cell death in CT26 colon cancer cells. The volcano plot identified 1138 differentially expressed genes (DEGs) in treated versus untreated CT26 cells, including 871 upregulated and 267 downregulated genes (Fig. 2a). Intersection analysis of DEGs with gene sets related to heat, calcium, and oxidative stress revealed 110 overlapping genes (Venn diagram, Fig. 2b). Protein–protein interaction (PPI) networks were constructed using the STRING database via Cytoscape software (Fig. 2c), and subsequent CytoHubba analysis identified the top-degree hub genes, *Jun, Hspa1b, Hsp90aa1, Fos, Cd4, Ptgs2, Hsp90b1, Cdh1, Egr1*, and *mt-Nd5*, which are strongly associated with ERS, mitochondria function, and necroptosis. Notably, 57 of the 110 DEGs directly intersected with gene sets for ERS, mitochondria, and necroptosis (Fig. 2d). Kyoto Encyclopedia of Genes and Genomes (KEGG) pathway enrichment analysis further demonstrated significant enrichment in pathways related to herpes simplex virus 1 infection, cell cycle, and protein processing in the ER, indicating the activation of diverse cell death related pathways (Fig. 2e). Given the established roles of intracellular ERS and mitochondrial dysfunction in triggering cell death, these findings collectively suggest that the combination of Ca^2+^/H_2_O_2_ co-incubation and mild hyperthermia induces necroptosis in CT26 cells by imposing them with multiple stresses, including Ca^2+^ overload, oxidative stress, and heat shock.

**Figure 3.**
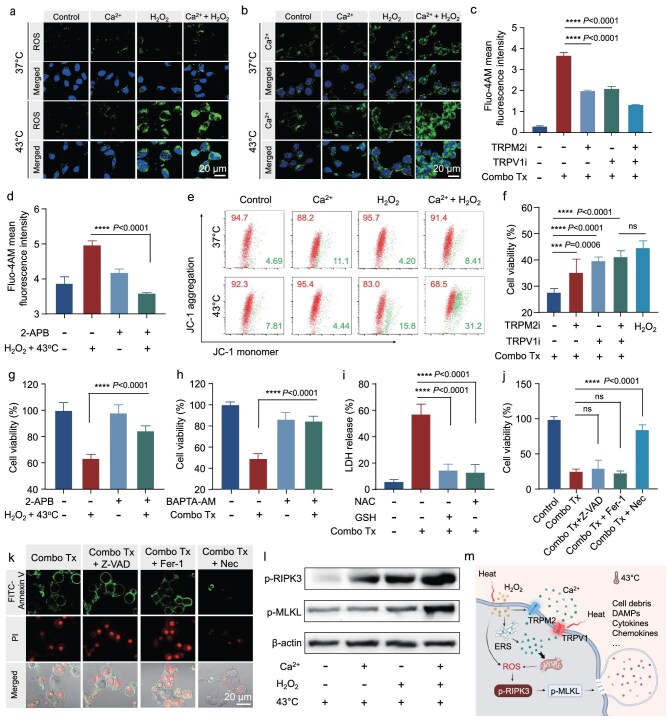
Ca^2+^/H_2_O_2_ co-incubation synergizes with subtherapeutic hyperthermia to induce cell necroptosis. (a and b) Confocal images of intracellular ROS generation (a) and Ca^2+^ content (b) of CT26 cells with indicated treatments. (c) Flow cytometry analysis of intracellular Ca^2+^ contents of CT26 cells treated with Ca^2+^/H_2_O_2_ co-incubation at 43°C in the presence and absence of TRPM2 inhibitor and TRPV1 inhibitor. The combination treatment of Ca^2+^/H_2_O_2_ co-incubation at 43°C was abbreviated as ‘Combo Tx’ in the figures for simplicity. (d) Flow cytometry analysis of intracellular Ca^2+^ contents of CT26 cells with indicated treatments. (e) Flow cytometry analysis of JC-1-stained CT26 cells with indicated treatments. (f) Cell viability of CT26 cells treated with the Combo Tx in the presence or absence of TRPM2 inhibitor and TRPV1 inhibitor. (g) Cell viability of CT26 cells with indicated treatments. (h) Cell viability of CT26 cells treated with the Combo Tx in the presence and absence of BAPTA-AM. (i) LDH release assay of CT26 cells treated with the Combo Tx in the presence and absence of GSH and NAC. (j and k) Relative cell viabilities (j) and confocal images (k) of CT26 cells treated with Combo Tx in the presence and absence of indicated molecular inhibitors. (l) Western blotting detection of p-RIPK3 and p-MLKL expression levels in CT26 cells with indicated treatments. (m) Schematic illustration showing the mechanism by which Ca^2+^/H_2_O_2_ co-incubation synergizes with subtherapeutic hyperthermia to induce cell necroptosis. Data are presented as the mean ± SD. Statistical analysis was performed using one-way analysis of variance. (****P < 0.0001, ***P < 0.001, **P < 0.01, *P < 0.05, ns: not significant).

To elucidate the mechanistic basis of this combinatorial effect, we then measured intracellular ROS and Ca^2+^ dynamics. Confocal microscopy and flow cytometry using 2′-7′-dichlorofluorescein diacetate revealed substantially elevated ROS levels in combination-treated cells compared to controls (Fig. 3a and Fig. S8). In contrast, CT26 cells treated by H_2_O_2_ (2 mM) incubation at 43°C for 30 min exhibited moderately increased intracellular ROS, while the other treatments showed negligible disturbance in intracellular ROS levels in CT26 cells. These results indicate that mild hyperthermia significantly enhances cellular H_2_O_2_ uptake, thereby synergizing with extracellular Ca^2+^ exposure to further increase intracellular ROS levels.

We then investigated the impacts of combinational treatment on intracellular Ca^2+^ concentrations, as both heat and oxidative stress are known to influence intracellular Ca^2+^ transients via promoting extracellular Ca^2+^ influx and intracellular Ca^2+^ release from the storages (e.g., ER) via distinct mechanisms [36–38]. Fluo-4 AM staining showed that CT26 cells with H_2_O_2_ incubation alone (using Ca^2+^-free medium) at both 37°C and 43°C exhibited increased Ca^2+^ concentration, suggesting intracellular oxidative stress amplification would promote Ca^2+^ release from ER (Fig. 3b and Fig. S9). Similarly, Ca^2+^ treatment at 43°C induced significant increase of intracellular Ca^2+^ compared to that incubated at 37°C, indicating activation of temperature-sensitive calcium channels in the plasma membrane. Notably, cells incubated with Ca^2+^ and H_2_O_2_ under 43°C showed the maximal intracellular Ca^2+^ accumulation, indicating the synergy of such combination treatment in promoting intracellular Ca^2+^ overload through concurrent activations of plasma membrane calcium channels and intracellular Ca^2+^ storage organelles.

Next, flow cytometry results revealed that the addition of JNJ-28583113 and SB-705498, targeting the oxidative stress-sensitive calcium channel (transient receptor potential melastatin 2, TRPM2) and the heat-sensitive calcium channel (transient receptor potential vanilloid 1, TRPV1) on the plasma membrane, respectively, partially suppressed the combination treatment (Combo Tx: Ca^2+^ + H_2_O_2_ under 43°C)-induced increase in intracellular Ca^2+^ levels (Fig. 3c). The addition of 2-aminoethyl diphenylborinate (2-APB), an inositol triphosphate receptor (IP3R) inhibitor that restricts Ca^2+^ release from the ER, completely abolished the combined mild hyperthermia and H_2_O_2_-cotreatment-induced cytoplasmic Ca^2+^ elevation as shown by flow cytometry (Fig. 3d). Western blotting further revealed that CT26 cells receiving the combination treatment showed markedly increased expression of CCAAT/enhancer binding proteins homologous protein/growth arrest and DNA damage inducible gene 153 (CHOP/GADD153) and glucose-regulated protein 78/immunoglobulin heavy chain binding protein (GRP78/Bip), two classic ERS markers (Fig. S10). These results confirm that amplification of intracellular oxidative stress promotes Ca^2+^ release from ER via inducing ERS, aligning with prior reports [39,40]. Collectively, these findings demonstrate that mild hyperthermia synergizes with Ca^2+^/H_2_O_2_ co-incubation to increase intracellular Ca^2+^ through promoting both extracellular Ca^2+^ influx via TRPM2 and TRPV1 channels, as well as intracellular Ca^2+^ release from the ER.

Given that mitochondria are a primary target of intracellular oxidative stress amplification [41], MitoSOX staining revealed a significant burst of mitochondrial ROS in CT26 cells following the combination treatment (Fig. S11). Accordingly, flow cytometry using the JC-1 probe showed substantial mitochondrial depolarization in CT26 cells with the combination treatment (Fig. 3e and Fig. S12), consistent with established evidence that intracellular ROS accumulation and Ca^2+^ overload induce mitochondrial dysfunction and regulate cell viability [23,42]. Consequently, blocking extracellular Ca^2+^ influx with JNJ-28583113 and SB-705498 significantly reversed combination treatment-induced cell death (Fig. 3f). Similarly, inhibiting ER Ca^2+^ release with 2-APB markedly prevented cell death in CT26 cells treated with H_2_O_2_ plus mild hyperthermia (Fig. 3g). Pretreatment with 1,2-bis(2-aminophenoxy)ethane-*N,N,N′,N′*-tetraacetic acid acetoxymethyl ester (BAPTA-AM), a cell-permeable Ca^2+^ chelator that removes intracellular free Ca^2+^, protected CT26 cells from Ca^2+^ overload and subsequent death (Fig. 3h and Fig. S13). Moreover, LDH release assays showed that ROS scavengers glutathione (GSH) and *N*-acetyl-L-cysteine (NAC) significantly attenuated the lytic death induced by the combination treatment (Fig. 3i and Fig. S14). Collectively, these results demonstrated that the Ca^2+^/H_2_O_2_ co-incubation under mild hyperthermia potently induces lytic cancer cell death through intracellular Ca^2+^ overload and oxidative stress amplification.

To elucidate cell death mechanisms, we employed inhibitors targeting distinct PCD pathways. Cell viability assay showed that while only 25.9% of combination-treated CT26 cells survived, co-treatment with the necroptosis inhibitor necrosulfonamide increased the relative cell viability to 85.6% (Fig. 3j and Fig. S15). In contrast, the pan-caspase inhibitor Z-VAD-FMK blocking pyroptosis/apoptosis and ferroptosis inhibitor ferrostatin-1 showed negligible effects on the viability of treated CT26 cells. Confocal microscopic observation further confirmed that only necrosulfonamide effectively suppressed the plasma membrane blebbing in treated CT26 cells (Fig. 3k). Furthermore, western blotting assay showed that the combination treatment resulted in significantly increased phosphorylation of RIPK3 and MLKL (Fig. 3l), two important proteins responsible for regulating necroptosis of cells by promoting the oligomerization of MLKL and its subsequent translocation into the plasma membrane to induce cell swelling and membrane rupture [43–45]. In addition, confocal microscopic observation showed hallmark immunogenic cell death features: nuclear high mobility group box 1 protein release and surface calreticulin expression (Figs S16 and S17). Finally, flow cytometry demonstrated that cell debris from CT26 cells subjected to Ca^2+^/H_2_O_2_ co-incubation under mild hyperthermia was most effective in promoting the maturation of BMDCs (Fig. S18), indicating the high immunogenicity of the necroptotic cancer cells.

Taken together, these results demonstrate that mild hyperthermia markedly enhances cellular H_2_O_2_ entry, likely by enhancing plasma membrane fluidity, thereby concurrently promoting extracellular Ca^2+^ influx through oxidative stress- and heat-sensitive plasma membrane channels. In the meanwhile, the mild hyperthermia treatment together with H_2_O_2_ exposure would trigger ERS and thus ER Ca^2+^ release via IP3R activation. The resultant intracellular Ca^2+^ overload induces mitochondrial damages and in turn promotes endogenous ROS generation, which together with exogenous H_2_O_2_ exposure drives necroptosis via activation of RIPK3–MLKL signaling axis (Fig. 3m). Together with previous reports that tumor cells are more sensitive to mild hyperthermia and ROS exposure [35,46], Ca^2+^/H_2_O_2_ co-incubation under mild hyperthermia therefore could selectively induce immunogenic necroptosis in cancer cells.

### *In vivo* TNIP-mediated HIP treatment and biocompatibility evaluation

**Figure 4.**
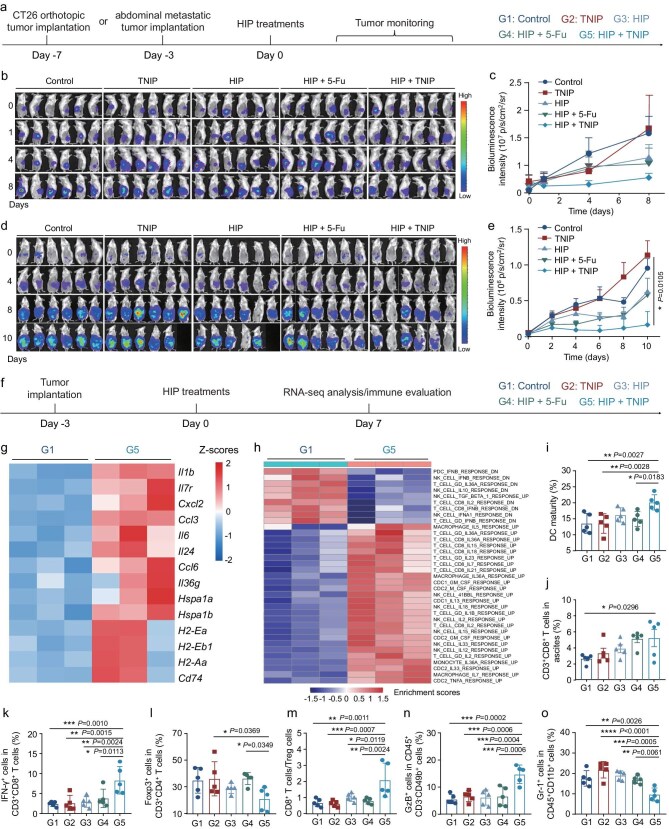
*In vivo* TNIP-based HIP treatment elicited antitumor immunity. (a) The experimental schedule of TNIP-based HIP treatment in orthotopic CT26 tumor models and abdominal metastatic Luc-CT26 tumor model. (b) Bioluminescence imaging of mice post indicated treatments (*n* = 6). (c) Semi-quantitative analysis of bioluminescence intensity of mice post indicated treatments based on the images shown in panel (b). (d) Bioluminescence imaging of mice with indicated treatments (*n* = 6). (e) Semi-quantitative analysis of bioluminescence intensity of mice post indicated treatments based on the images shown in panel (d). (f) Schematic illustration of the experimental schedule for evaluating the combination treatment-induced antitumor immunity. (g) Heat map of selected immune-function-related genes. (h) A heat map showing the enrichment scores of gene set functions in GSVA. (i) DC maturation status in the mesenteric lymph nodes of mice with indicated treatments. (j–o) The frequencies of CD3^+^CD8^+^ T cells (j), CD3^+^CD8^+^IFN-γ^+^ T cells (k), CD3^+^CD4^+^Foxp3^+^ Tregs (l), CD8^+^ T cells/Tregs ratios (m), GzB^+^ NK cells (n), and CD45^+^CD11b^+^Gr-1^+^ MDSCs (o) in peritoneal lavage fluids of mice following indicated treatments. Data are presented as the mean ± SD. Statistical analysis was performed using one-way analysis of variance. (****P < 0.0001, ***P < 0.001, **P < 0.01, and *P < 0.05).

Motivated by the superior stability and selective necroptosis-inducing capacities of concurrent Ca^2+^/H_2_O_2_ exposure under mild hyperthermia (Fig. S19), we then evaluated the potential of utilizing the Ca^2+^ and H_2_O_2_ solution as the TNIP for HIP treatment of intraperitoneal tumors (Fig. 4a). Colorectal CT26 orthotopic transplantation tumor models were first established by injecting luciferase-transfected CT26 (Luc-CT26) cells into the colon wall of Balb/c mice. On the seventh day after tumor implantation, 30 Luc-CT26 orthotopic tumor-bearing mice were randomized into 5 groups for the following treatments: group 1, no-treatment group (control); group 2, intraperitoneal perfusion with TNIP (8 mM Ca^2+^, 2 mM H_2_O_2_); group 3, HIP with 0.9% NaCl solution; group 4, HIP with 5-Fu (15 mg kg^−1^); and group 5, HIP with TNIP (8 mM Ca^2+^, 2 mM H_2_O_2_). The fluid flow rate, fluid temperature, and treatment time for related perfusion operation were kept at 3 mL min^−1^, 43°C, and 30 min, respectively. By recording the bioluminescence signals, it was found that TNIP-based HIP therapy exhibited significantly better tumor suppression efficacy than the standard 5-Fu-based HIP treatment, which is commonly used in the clinical treatment of primary and metastatic colorectal tumors [47] (Fig. 4b and c). Noticeably, one of six mice receiving TNIP-based HIP treatment achieved complete remission. Obviously prolonged median survival of such TNIP-based HIP treatment versus 5-Fu-based HIP treatment was observed (Fig. S20a). No significant body weight loss was observed in the TNIP-based HIP treatment group (Fig. S20b). These results collectively indicate that the TNIP-based HIP treatment could effectively suppress the growth of orthotopic CT26 tumors without imposing significant side effects at the performed dosage.

Next, we investigated the potency of the TNIP-based HIP treatment against abdominal metastatic colon tumors, which is the most widely used situation for HIPEC treatment [48–50]. To this end, 30 abdominal metastatic CT26 tumor-bearing Balb/c mice were established by intraperitoneal injection of 1 × 10^6^ Luc-CT26 cells 3 days prior to receiving the same treatments as mentioned above. The whole-body bioluminescence imaging results showed that the TNIP-based HIP treatment exhibited the most effective tumor inhibitory effect (Fig. 4d and e), and two of six mice in this group were cured without obvious recurrence observed over 50 days (Fig. S21a). In marked contrast, the 5-Fu-based HIP treatment only slightly suppressed the growth of abdominal metastatic tumor nodules, and the other treatments showed negligible tumor inhibitory effect. In addition, no significant body weight loss was observed in the mice with the aforementioned treatments (Fig. S21b).

To evaluate the broad-spectrum therapeutic ability of the TNIP-based HIP treatment, we tested its therapeutic efficacy in murine ID8 epithelial ovarian cancer, another type of abdominal tumors that commonly needs HIPEC treatment when diagnosed at the advanced stage in clinic [51–53]. To this end, an abdominal metastatic epithelial ovarian tumor model was established by injecting luciferase-expressing ID8 (Luc-ID8) cells into the abdominal cavity of C57BL/6 mice (Fig. S22a). On day 3 after the tumor inoculation, five groups of Luc-ID8 bearing mice (*n* = 5) received the following treatments: group 1, untreated; group 2, intraperitoneal perfusion with TNIP (8 mM Ca^2+^, 2 mM H_2_O_2_); group 3, HIP with 0.9% NaCl solution; group 4, HIP with cisplatin (3 mg kg^−1^); group 5, HIP with TNIP (8 mM Ca^2+^, 2 mM H_2_O_2_). The perfusion operation was kept at a fluid flow rate of 3 mL min^−1^, and 43°C for 30 min. The whole-body bioluminescence imaging showed that the TNIP-based HIP treatment effectively inhibited the progression of abdominal Luc-ID8 metastasis (Fig. S22b and S22c). In sharp contrast, cisplatin-based HIP treatment, which is commonly used in the clinical treatment of ovarian cancer [6], only moderately delayed the progression of the abdominal Luc-ID8 metastasis. Taken together, these results verify the superiority of the TNIP-based HIP treatment in suppressing the progression of both abdominal orthotopic tumors and diffuse metastatic nodules.

We subsequently evaluated the biocompatibility of TNIP-based HIP treatment. Biochemical blood analysis revealed that 5-Fu-HIP treatment using the same experimental parameters as mentioned above led to significantly increased serum levels of alanine aminotransferase, aspartate transaminase, and urea, along with the reduced alkaline phosphatase (ALP) level at 24 h post-treatment (Fig. S23). In contrast, the TNIP-based HIP treatments only decreased the serum ALP level at 24 h post-treatment. All affected parameters were back to normal 7 and 14 days after treatment in both groups. In addition, routine blood test demonstrated minimal perturbations in red blood cell count, hemoglobin content, hematocrit, mean corpuscular volume, mean corpuscular hemoglobin, mean corpuscular hemoglobin concentration, and platelet count. These results collectively suggest that TNIP-based HIP treatment exhibited no significant systemic toxicity at the administered dosage.

### TNIP-based HIP treatment primes antitumor immunity

We next investigated the potency and detailed mechanism of the TNIP-based HIP treatment in triggering antitumor immunity via bulk transcriptome sequencing analysis (Fig. 4f). Transcriptome sequencing results revealed 307 DEGs in malignant ascites from TNIP-HIP-treated mice bearing abdominal metastatic CT26 tumors compared to control group counterparts, with 111 upregulated and 196 downregulated DEGs (Fig. S24). KEGG pathway enrichment analysis demonstrated significant activation of critical innate and adaptive immune related pathways including cytokine–cytokine receptor interaction, tumor necrosis factor (TNF) signaling, antigen processing and presentation, and chemokine signaling pathway in TNIP-HIP-treated mice (Fig. S25). Notably, we observed substantial upregulation of pro-inflammatory cytokines (e.g., *Il1b, Il6, Il24, Il36g*, and *Il17r*) and chemokines (e.g., *Cxcl2, Ccl3* and *Ccl6*) known to initiate antitumor immunity by recruiting and activating immune effector cells including T cells, natural killer (NK) cells, and DCs (Fig. 4g). Enhanced tumor antigen presentation was evidenced by upregulated DEGs in antigen processing pathways (e.g., *Hspa1a, Hspa1b, H2-Ea, H2-Eb1, H2-Aa* and *Cd74*), suggesting improved antigen-presenting cell (APC)-mediated T-cell activation. Additionally, gene set variation analysis (GSVA) using the immunologic gene set collection ‘m7.all.v2026.1.Mm.symbols’ from the Molecular Signatures Database identified 35 gene sets significantly correlated with TNIP-HIP treatment (Fig. 4h). These gene sets were linked to the responses of various immune cells, including NK cells, DCs, and T cells, to diverse immunostimulatory cytokines (e.g., interferon-β (IFN-β), IFN-α1, interleukin-36A (IL-36A), IL-2, IL-15, IL-18, IL-23, IL-7, IL-21, and IL-33). Together with the upregulation of immune function-related DEGs shown in Fig. 4g, these findings characterize a reprogramming of the TME toward an antitumor immune phenotype driven by TNIP-HIP treatment.

After that, flow cytometry analysis was performed to verify the ability of TNIP-based HIP treatment to elicit antitumor immune responses. To this end, five groups of mice with abdominal metastatic CT26 tumors (*n* = 5) received the same treatments as mentioned in Fig. 4a. Mice from different groups were sacrificed 7 days post-treatment, with their malignant ascites and peritoneal lymph nodes collected for preparing single cell suspensions. TNIP-HIP-treated mice exhibited pale red-to-colorless ascites, contrasting with the blood-red ascites of untreated mice, which is a visual indicator of reduced intraperitoneal tumor burden and hemorrhagic ascites (Fig. S26). Flow cytometric analysis results revealed significantly enhanced DC maturation in peritoneal lymph nodes compared to other interventions (Fig. 4i and Fig. S27). In addition, the TNIP-based HIP treatment also remarkably increased the abundances of CD3^+^CD8^+^ T cells in ascites (Fig. 4j) and augmented their activation, as evidenced by elevated intracellular IFN-γ^+^ T-cell population (Fig. 4k and Fig. S28). Together with its ability to downregulate the abundances of immunosuppressive regulatory T cells (Tregs) (Fig. 4l and Fig. S29), such TNIP-based HIP treatment resulted in maximum increase of CD3^+^CD8^+^ T cells/Tregs ratios (Fig. 4m), an important sign of activation of host’s adaptive antitumor immunity. Further analysis demonstrated that the TNIP-based HIP treatment could also activate innate immunity via activation of NK cells (CD45^+^CD3^−^CD49b^+^GzB^+^) (Fig. 4n and Fig. S30), reduction of immunosuppressive myeloid-derived suppressor cells (MDSCs, CD45^+^CD11b^+^Gr-1^+^) (Fig. 4o), and repolarization of tumor-associated macrophages to pro-inflammatory M1 type macrophages (Fig. S31) in the ascites. Taken together, these findings demonstrate that the TNIP-based HIP treatment is able to elicit both innate and adaptive antitumor immunity.

### TNIP-based HIP treatment synergizes with ICB immunotherapy for durable tumor control

**Figure 5.**
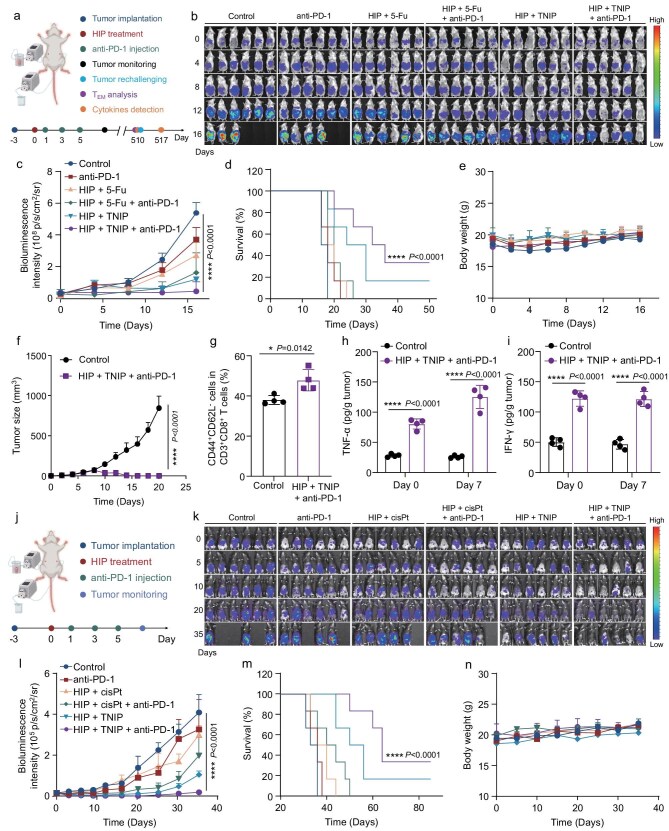
Tumor inhibitory efficacy of combined TNIP-based HIP and ICB immunotherapy. (a) Schematic illustration of the experimental schedule for the combination treatment in Luc-CT26 abdominal metastatic tumor model (*n* = 6). (b) Bioluminescence imaging of mice post indicated treatments. (c) Semi-quantitative analysis of bioluminescence intensity of mice post different treatments as shown in the images in panel (b). (d and e) Survival rate (d) and body weights (e) of mice receiving indicated treatments. (f) The tumor growth curves of rechallenged CT26 tumor. (g) Proportions of T_EM_ in peripheral blood from indicated mice right before CT26 tumor rechallenge. (h and i) Cytokine levels including TNF-α (h) and IFN-γ (i) in sera from mice isolated before and 7 days after CT26 tumor rechallenge. (j) Schematic illustration of the experimental schedule for the combination treatment in Luc-ID8 abdominal metastatic tumor model (*n* = 6). (k) Bioluminescence imaging of mice post indicated treatments. (l) Semi-quantitative analysis of bioluminescence intensity of mice post different treatments as shown in the images in panel (k). (m and n) Survival rate (m) and body weights (n) of mice receiving indicated treatments. (****P < 0.0001, ***P < 0.001, **P < 0.01, and *P < 0.05).

Motivated by the efficacy of TNIP-based HIP treatment in eliciting potent antitumor immune responses, we therefore investigated its potential synergy with anti-programmed death receptor 1 (anti-PD-1) immunotherapy for enhanced tumor control (Fig. 5a). Six groups of Balb/c mice with abdominal metastatic Luc-CT26 tumors received the following treatments: group 1, untreated; group 2, anti-PD-1 injection; group 3, HIP with 5-Fu; group 4, HIP with 5-Fu and anti-PD-1 injection; group 5, HIP treatment with TNIP; group 6, HIP treatment with TNIP and anti-PD-1 injection. The dosages of 5-Fu and TNIP were the same as mentioned above, and anti-PD-1 was intravenously injected at a dose of 1 mg kg^−1^ per injection at 1, 3, and 5 days after the HIP treatment. Whole-body bioluminescence imaging revealed that the therapeutic potency of the TNIP-HIP treatment combined with anti-PD-1 immunotherapy was markedly enhanced with the prolonged median survival time of 32 days, and two of six mice were cured (Fig. 5b–d). Conversely, anti-PD-1 provided minimal augmentation to 5-Fu-based HIP treatment. No treatment-related weight loss was observed across groups (Fig. 5e), indicating favorable safety profiles at tested doses. These results collectively demonstrate effective synergy between TNIP-based HIP treatment with anti-PD-1 immunotherapy against the progression of CT26 abdominal metastases.

Next, we investigated the long-term immune memory induced by TNIP-based HIP treatment combined with anti-PD-1 immunotherapy. In a standard tumor rechallenge assay, four mice bearing abdominal metastatic CT26 tumors that had been cured by the combination treatment completely rejected subcutaneous CT26 tumors inoculated at 510 days post first round treatment (Fig. 5f and Fig. S32). In contrast, healthy mice receiving the same tumor inoculation exhibited rapid tumor growth. Flow cytometry analysis of peripheral blood collected right before tumor rechallenge showed a significantly higher proportion of effector memory T cells (CD3^+^CD8^+^CD44^+^CD62L^−^, T_EM_) in the cured mice compared to healthy controls (Fig. 5g and Fig. S33). Moreover, enzyme-linked immunosorbent assay (ELISA) revealed markedly elevated serum levels of TNF-α and IFN-γ, two effector cytokines associated with immune memory, in cured mice both before and 7 days after tumor inoculation, relative to healthy mice (Fig. 5h and i). Collectively, these findings demonstrate that the combination of HIP-mediated TNIP and anti-PD-1 elicits robust, long-term immune memory for durable tumor control.

The synergistic therapeutic efficacy of the TNIP-based HIP treatment and anti-PD-1 immunotherapy was further confirmed in an ID8 abdominal metastatic tumor model (Fig. 5j). At day 3 after intraperitoneal tumor inoculation, six groups of abdominal metastatic Luc-ID8-bearing C57BL/6 mice received the following treatments: group 1, untreated; group 2, intravenous anti-PD-1 injection; group 3, HIP with cisplatin; group 4, HIP with cisplatin and anti-PD-1 injection; group 5, HIP with TNIP; group 6, HIP with TNIP and anti-PD-1 injection. The doses of corresponding agents were same as those mentioned above. Consistent with CT26 results, bioluminescence quantification demonstrated superior tumor control in TNIP-HIP treatment combined with anti-PD-1 immunotherapy group (Fig. 5k and l) with median survival extending to 64 days compared to TNIP-HIP monotherapy (Fig. 5m). Mirroring previous observations, anti-PD-1 provided negligible enhancement to cisplatin-HIP efficacy. All regimens maintained stable body weights (Fig. 5n), reinforcing the combined therapy’s safety profile. These results confirm broad applicability of TNIP-HIP/anti-PD-1 synergy across tumor models.

### TNIP-based HIP treatment in human PDOs

**Figure 6.**
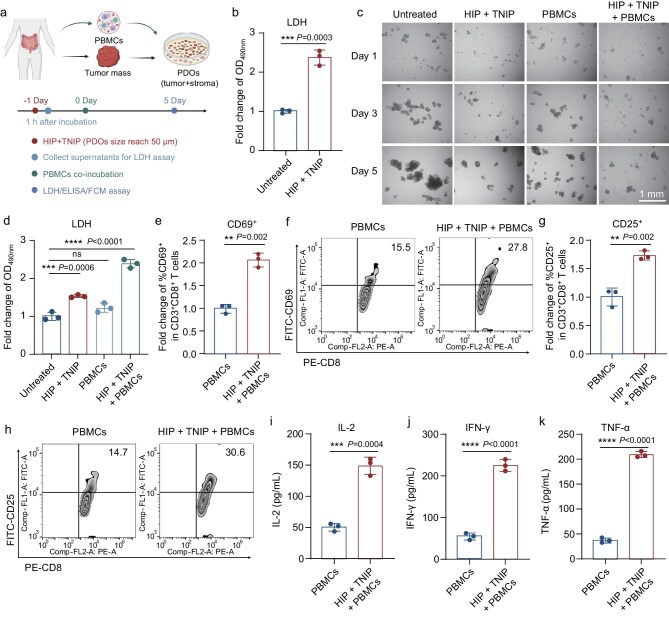
Therapeutic effect of TNIP-based HIP on human patient-derived colon cancer organoids. (a) A schematic diagram illustrating the process of establishing PDOs for evaluating TNIP-based HIP treatment-induced PDO killing and immune activation. (b) LDH assay results of PDOs with indicated treatments. (c) Optical microscopic images of PDOs on days 1, 3, and 5 post indicated treatments. (d) LDH assay results of PDOs with indicated treatments on 5 days post-treatments. (e and f) Corresponding quantification results (e) and representative flow cytometric zebra plots (f) of CD69^+^CD3^+^CD8^+^ T cell population in PBMCs with indicated treatments. (g and h) Corresponding quantification results (g) and representative flow cytometric zebra plots (h) of CD25^+^CD3^+^CD8^+^ T cell population in PBMCs with indicated treatments. (i–k) Secretion levels of IL-2 (i), IFN-γ (j), and TNF-α (k) in the PBMCs with indicated treatments. Data are presented as the mean ± SD. Statistical analysis was performed using *t*-test analysis. (****P < 0.0001, ***P < 0.001, **P < 0.01, *P < 0.05, ns: not significant).

PDOs have recently been recommended as a reliable therapeutic screening platform because they can reconstruct phenotypic and genetic characteristics of the original tumor lesions in patients, surpassing conventional methods like two-dimensional cell culture and murine models [54]. We therefore investigated the antitumor efficacy of the TNIP-based HIP treatment using PDOs established from advanced colorectal cancer samples (Fig. 6a). It was shown that PDOs (50 μm in diameter) treated with H_2_O_2_ (2 mM) and CaCl_2_ (8 mM) at 43°C for 1 h exhibited significantly increased release of LDH compared to untreated PDOs (Fig. 6b), indicating the robust therapeutic efficacy of the TNIP-based HIP treatment against human cancer cells.

Furthermore, co-culturing PDOs with autologous peripheral blood mononuclear cells (PBMCs) has been validated as a platform for inducing tumor-reactive T cells to assess the capacity of T-cell-mediated specific tumor cell killing [55]. A co-culture model of PDOs and autologous PBMCs at a 4:1 effector-target ratio of (2 × 10^5^ of PBMCs) was established for evaluating the immune activation capacity of TNIP-based HIP treatment. Microscopic examination and LDH assays simultaneously demonstrated that the addition of PBMCs significantly enhanced the suppression effect of TNIP-based HIP treatment on the growth of PDOs (Fig. 6c and d). Moreover, flow cytometric analysis demonstrated that TNIP-based HIP treatment resulted in significant upregulation of CD69 and CD25 molecules on the surface of CD45^+^CD3^+^CD8^+^ T cells in PBMCs, demonstrating the sustained activation of effector T cells (Fig. 6e–h and Figs S34 and S35). Additionally, PBMCs co-incubated with TNIP-based HIP-treated PDOs also exhibited markedly increased secretion of IL-2, TNF-α, and IFN-γ, which were tightly associated with effector T-cell activation (Fig. 6i–k). Collectively, these results demonstrate that TNIP-based HIP treatment strategy could not only directly kill PDOs, but also markedly activate autologous effector T cells to amplify PDO suppression.

## DISCUSSION

HIPEC has been established as a first-line locoregional treatment modality for diverse peritoneal malignancies due to its superior pharmacokinetic advantages and improved patient compliance compared to corresponding systemic chemotherapy. However, current HIPEC protocols continue to face challenges with high rates of chemotherapy-related adverse effects, including bone marrow suppression, renal dysfunction, and intraperitoneal infection. In this study, we demonstrate that co-incubation of cancer cells with Ca^2+^ and H_2_O_2_ under short-term mild hyperthermia synergistically promoted intracellular Ca^2+^ overload via simultaneously activating Ca^2+^-influxing channels and triggering IP3R-dependent ER Ca^2+^ release, subsequently amplifying the intracellular oxidative stress. As a result, such treatment strategy induced potent immunogenic necroptosis for different types of cancer cells via activating the RIPK3–MLKL signaling pathway. Notably, we discovered that the mild hyperthermia treatment of cells with Ca^2+^/H_2_O_2_ co-incubation in a concentration range being toxic to cancer cells showed much less impact to viabilities of normal cells. Given that both Ca^2+^ and H_2_O_2_ are endogenous chemicals well-tolerated by the body, and H_2_O_2_ is prone to be enzymatically decomposed by endogenous catalase, our proposed HIP treatment using a binary Ca^2+^/H_2_O_2_ solution as the tumor-sensitive necroptosis-inducing perfusate holds great potential for avoiding the chemotherapy-related side effects in clinical HIPEC. Furthermore, the inherent disinfectant properties of H_2_O_2_ enable our TNIP-mediated HIP treatment to substantially reduce infection risks during surgical procedures.

Recent evidence indicates that HIPEC hyperthermia can moderately enhance the host’s antitumor immunity, potentiating the effects of anti-PD-L1 therapy to collectively inhibit ovarian tumor growth [6]. Considering the well-characterized immunostimulatory capacity of necroptotic cancer cells, TNIP-based HIP treatment demonstrated superior ability over conventional HIPEC in activating both innate and adaptive antitumor immunity. Consequently, this TNIP-based HIP treatment exhibited superior tumor suppression efficacy across multiple tumor models compared to 5-Fu- and cisplatin-based HIPEC treatments, particularly when combined with anti-PD-1 immunotherapy. This TNIP-based HIP treatment also demonstrated superior antitumor and immune-activating capacities in autologous PBMCs-supplemented human PDOs. Additionally, attributed to the potency of necroptotic cancer cells to induce long-term immune memory by functioning as cancer vaccines [56], TNIP-based HIP treatment induced potent long-term immune memory, and holds promise to function as a neoadjuvant strategy for postoperative ICB immunotherapy, synergistically preventing tumor recurrence.

In summary, this study depicts that Ca^2+^/H_2_O_2_ co-incubation under mild hyperthermia is capable of selectively inducing necroptosis in cancer cells by disrupting intracellular calcium and oxidative stress homeostasis, while largely preserving viabilities of normal cells within an appropriate concentration range. Utilizing the binary Ca^2+^ and H_2_O_2_ solution as a TNIP, we establish an effective yet chemo-free hyperthermic intraperitoneal immunotherapy regimen. Compared to chemotherapeutics used in conventional HIPEC whose cytotoxicities are not significantly affected by the temperature, our administered Ca^2+^/H_2_O_2_ perfusate with durable stability shows little toxicity at the physiological body temperature, especially upon diffusion into lower concentrations, thus imposing much less risk of sustained toxic side effects. Meanwhile, by triggering necroptosis of cancer cells and activating T-cell-mediated antitumor immunity, this approach not only achieves potent tumor suppression by itself, but also shows the potential to synergize with clinical immunotherapy for further improved therapeutic performance. Given that both CaCl_2_ and H_2_O_2_ solutions are already clinically approved for hypocalcemia treatment and wound cleaning, respectively, this immunogenic perfusate can be readily prepared at the bedside by simple dilution without additional manufacturing steps. Thus, TNIP-mediated HIP treatment represents a promising therapeutic alternative for managing peritoneal malignancies, with the potential to replace chemotherapy-based HIPEC currently used in clinical practice.

## MATERIALS AND METHODS

Detailed materials and methods can be found in the online supplementary file.

### Ethical statements

Balb/c and C57BL/6 mice were purchased from Laboratory Animal Center of Soochow University, and used by following the protocols approved by Laboratory Animal Center of Soochow University (SUDA20240724A01). Advanced colorectal cancer samples and autologous PBMCs were collected at Shanxi provincial cancer hospital following the protocols approved by the local Ethics Committee (YXYJ2022012). Written informed consent was obtained from patients.

## Supplementary Material

nwag321_Supplementary_data_clean_20260522
