# Supplementary material for: A chemo-free thermo-sensitive necroptosis-inducing perfusate to enable potent hyperthermic intraperitoneal immunotherapy
Source: Natl Sci Rev. 2026 May 28;13(14):nwag321. doi: 10.1093/nsr/nwag321 (PMC13382887; doi:10.1093/nsr/nwag321)
Supplement: nwag321_Supplementary_data_clean_20260522 [file nwag321_supplementary_data_clean_20260522.pdf]

A chemo-free thermo-sensitive necroptosis inducing perfusate to enable potent hyperthermic intraperitoneal immunotherapy

Yujie Zhu[1,#], Lin Zhang[2,#], Shiqi Yang[1,#], Chunjie Wang[1], Xiangyu Zhang[1], Ning Li[3], Zhuang Liu[1,], Liangzhu Feng[1,]**

Affiliations:

[1] Institute of Functional Nano & Soft Materials (FUNSOM), Jiangsu Key Laboratory for Carbon-Based Functional Materials & Devices, Biomedical Basic Research Center (BBRC) of Jiangsu Province, Engineering Research Center of RNA Medicine and Cell Therapy Technology, Ministry of Education, Soochow University, Soochow University, 199 Ren'ai Road, Suzhou, 215123, Jiangsu, PR China.

[2] Department of Obstetrics and Gynecology, The First Affiliated Hospital of Soochow University, Suzhou, China.

[3] Department of Radiation Oncology, National Cancer Center/National Clinical Research Center for Cancer/Cancer Hospital, Chinese Academy of Medical Sciences, Peking Union Medical College, Beijing 10021, China.

*Corresponding author. Email: zliu@suda.edu.cn, lzfeng@suda.edu.cn

Supplementary information:

MATERIALS AND METHODS

Materials: $CaCl_2$ and DCFH-DA were obtained from Sigma-Aldrich. H_2O_2 was purchased from Shanghai Peak Chemical Reagent Co., Ltd. LDH cytotoxicity assay kit, Fluo-4 AM calcium ion probe, and JC-1 mitochondrial membrane potential assay kit were purchased from Beyotime

Biotech Co., Ltd. Methyl thiazolyl tetrazolium (MTT) was purchased from Beijing Solarbio Science & Technology Co., Ltd. RPMI 1640 medium (no calcium nitrate) and DMEM high glucose medium were purchased from HyClone. Fetal bovine serum (FBS, BS-1105) was purchased from Inner Mongolia Opcel Biotechnology Co., Ltd. Fluorophore labeled antibodies for flow cytometric analysis were obtained from Biolegend and eBioscience. Anti-PD-1 was obtained from BioXcell. Collagenase I, collagenase IV, and hyaluronidase Grade I were purchased from Wuxi Lifes Biological Laboratory Equipment Co., Inc. Antibodies for flow cytometry assays including anti-CD11c-FITC (Biolegend, clone N418, Catalog: 117306), anti-CD80-APC (Biolegend, clone 16-10A1, Catalog: 104713), anti-CD86-PE (Biolegend, clone GL-1, Catalog: 105008), anti-CD3-FITC (Biolegend, clone 17A2, Catalog: 100204), anti-CD8a-PE (Biolegend, clone 53-6.7, Catalog: 100708), anti-CD4-APC (Biolegend, clone GK1.5, Catalog: 100412), anti-Foxp3-PE (Biolegend, clone MF-14, Catalog: 126404), anti-CD45-FITC (Biolegend, clone 30-F-11, Catalog: 103108), anti-CD3-PE (Biolegend, clone 145-2C11, Catalog: 100308), anti-CD49b-APC (Biolegend, clone HMa2, Catalog: 104516), anti-CD44-PE (Biolegend, clone IM7, Catalog: 103008), anti-CD62L-APC (Biolegend, clone MEL-14, Catalog: 104412), anti-CD69-FITC (Biolegend, clone H1.2F3, Catalog: 104505), anti-CD11b-PE (Biolegend, clone M1/70, Catalog: 101208), anti Gr-1-APC (Biolegend, clone RB6-8C5, Catalog: 108412), anti-IFN-γ-APC (Biolegend, clone XMG1.2, Catalog: 505810), anti-CD8a-PE-Cy7 (Biolegend, clone S18018E, Catalog: 162311), anti-CD3-Percp (Biolegend, clone 17A2, Catalog: 100287) were obtained from Biolegend as indicated and diluted at 1 : 300 for cell staining.

Cell experiments

Murine colon carcinoma CT26 cells, murine breast carcinoma 4T1 cells, human embryonic kidney HEK-293T cells, human cervical carcinoma HeLa cells, human gastric carcinoma HGC-27 cells, hepatoblastoma carcinoma HepG2 cells, murine embryonic fibroblast NIH-3T3 cells, and human umbilical vein endothelial HUVEC cells were purchased from the Cell Bank, Shanghai Institutes for Biological Sciences, Chinese Academy of Sciences. Murine ID8 ovarian epithelial cancer cells were obtained as a gift from the International Peace Maternity and Child Health Hospital, Shanghai Jiao Tong University. All cell lines were cultured in the standard incubator under 5% CO_2 at 37 ºC according to vendors' procedures.

To evaluate the cytotoxic effects of Ca^{2+}/H_2O_2 co-incubation under hyperthermia, CT26 cells were seeded in 96-well cell culture plates (1×10^4 cells) overnight, followed by being incubated with varying concentrations of Ca^{2+} and H_2O_2 at 37 °C or 43 ºC for 30 min. Cell viability was assessed using standard MTT assay and LDH release assay according to vendors' procedure. The same experimental procedures were applied on ID8, 4T1, HeLa, HGC-27, HepG2, NIH-3T3, BMDCs, HEK-293T, and HUVEC cells.

To observe the morphology evolution profiles of different cancer cells after Ca^{2+}/H_2O_2 co-incubation plus hyperthermia treatment, pre-seeded CT26 cells (1×10^5 cells) were incubated with the fresh RPMI 1640 containing varying concentrations of $CaCl_2$ (0, 8 mM) and H_2O_2 (0, 2 mM) at 37 or 43 °C for 30 min. Then, the treated CT26 cells were washed twice with PBS before being stained with FITC-Annexin V and propidium iodide (PI) for subsequent confocal microscopic observation (Zeiss, LSM 800).

To test the intracellular oxidative stress amplification, CT26 cells (1×10^5 cells) with the same treatments as mentioned above were washed twice with PBS before being incubated with DCFH-DA (10 μM, 30 min). Cells were sequentially fixed with 4% paraformaldehyde,

counterstained with DAPI (1 μg mL^{-1}), and imaged using confocal microscopy. Additionally, the intracellular oxidative stress levels of CT26 cells with same treatments as mentioned were also semiquantitative quantified via flow cytometric analysis (BD Accurit C6 Plus).

To investigate the intracellular Ca^{2+} concentration, CT26 cells with above mentioned treatment were washed twice with PBS before being incubated with Fluo-4 AM (2 μM, 30 min). After being fixed with 4% paraformaldehyde, counterstained with DAPI, these CT26 cells were subjected to confocal microscopic observation. Additionally, the intracellular Ca^{2+} concentration in CT26 cells with same treatments as mentioned above were also subjected to semi-quantitative flow cytometric analysis.

To test the mitochondria damage, CT26 cells with same treatments as mentioned above were washed twice with PBS, stained with JC-1 (5 μg mL^{-1}) for 30 min, and subjected to quantitative flow cytometric analysis by following the instructions in the kit.

To study the pathway of extracellular calcium ion influx following Ca^{2+}/H_2O_2 co-incubation plus hyperthermia treatment, CT26 cells were pre-seeded in 12-well cell culture plates (1×10^5 cells) overnight. Prior to treatment with Ca^{2+}/H_2O_2 (8 mM, 2 mM) and mild hyperthermia, CT26 cells were pretreated for 2 h with either JNJ-28583113 (an inhibitor of oxidative stress-sensitive calcium channel TRPM2, 10 μM) or SB-705498 (an inhibitor of heat-sensitive calcium channel TRPV1, 10 μM). Subsequently, cells were stained with Fluo-4 AM as described above for flow cytometric analysis. Additionally, the cell viability of CT26 cells pretreated with TRPV1 and TRPM2 were also subjected to MTT assay.

To study ER Ca^{2+} release during H_2O_2 incubation plus hyperthermia treatment, pre-seeded CT26 cells were first treated with 2-APB (100 μM) for 6 h before being subjected to H_2O_2 (2 mM) at 43 °C for 30 min. Later, these cells were stained with Fluo-4 AM as above

mentioned and analyzed via flow cytometry. Additionally, the cell viability of CT26 cells with same treatment was also subjected to MTT assay.

To investigate the impact of intracellular calcium levels on the occurrence of lytic cell death after Ca^{2+}/H_2O_2 co-incubation plus hyperthermia treatment, CT26 cells were pre-seeded in 96-well cell culture plates (1×10^4 cells) overnight. Before being treated with Ca^{2+}/H_2O_2 (8 mM, 2 mM) and mild hyperthermia, CT26 cells were pretreated with BAPTA-AM (2.5 μM) for 2 h. Cell viability was subsequently assessed using the standard MTT assay. Similarly, to investigate the role of ROS levels in the occurrence of lytic cell death under the same experimental conditions, CT26 cells were pre-seeded in 96-well cell culture plates (1×10^4 cells) overnight. Before being treated with Ca^{2+}/H_2O_2 (8 mM, 2 mM) and mild hyperthermia, CT26 cells were pretreated with GSH (5 mM) or NAC (5 mM) for 2 h, Subsequently, the viability of treated cells was determined via the standard MTT assay.

To investigate programmed cell death pathways in CT26 cells under Ca^{2+}/H_2O_2 co-incubation with hyperthermia, cells pre-seeded in 96-well cell culture plates (1×10^4 cells) were pretreated for 2 h with necrosulfonamide (5 μM), Z-VAD (50 μM), and ferrostatin-1 (10 μM) prior to the treatment of Ca^{2+}/H_2O_2 co-incubation and mild hyperthermia as mentioned above. Cell viability and morphological changes were assessed using previously described methodologies.

For western blotting assay, CT26 cells treated as described above were harvested, a and protein samples were extracted using commercial radioimmunoprecipitation (RIPA) buffer supplemented with protease inhibitors. Then, the protein samples were separated by sodium dodecyl sulfate polyacrylamide gel electrophoresis (SDS-PAGE), transferred to polyvinylidene fluoride (PVDF) membrane, and incubated with anti-pRIPK3 (1: 1000 dilution) and anti-pMLKL

(1: 1000 dilution) antibodies and corresponding secondary antibodies before being imaged under chemiluminescence imaging system (Amersham Imager 600, GE) according to the standard protocol.

To test the cellular ATP release, the supernatants of CT26 cells with the same treatment as aforementioned above were collected, and ATP concentrations were quantified using a commercial ATP assay kit. CT26 cells subjected to the aforementioned treatments were stained with anti-calreticulin (catalog: ab2907) or anti-HMGB1 primary antibodies (catalog: 70-ab40050-100), and their corresponding fluorescent secondary antibodies (catalog: 111-545-003, Jackson) for evaluating the CRT expression and HMGB1 release via the microscopic observation and flow cytometry, respectively.

To test the influence of tumor cell debris generated from different treatments on DCs maturation, BMDCs from C57BL/6 mice were prepared by according to an established method[1]. Then, BMDCs were seeded in 24-well nontreated plates (1×10^6 cells) and then incubated with tumor cell debris generated from CT 26 cells with above mentioned treatments or lipopolysaccharide (LPS) for 15 hours before being stained with fluorescent antibodies against CD11c, CD86, and CD80 for 30 min and analyzed via the flow cytometry.

Animal experiments

Balb/c and C57BL/6 mice were purchased from Laboratory Animal Center of Soochow University, and used by following the protocols approved by Laboratory Animal Center of Soochow University. To inoculate Luc-CT26 orthotopic transplantation tumor models, mice were anesthetized and Luc-CT26 cells (1×10^6) suspended in 10 μL PBS were injected into the wall of the cecum after an incision was made in the abdomen, followed by sequential suturing of

the abdominal muscle and external skin. To inoculate Luc-CT26 and Luc-ID8 abdominal metastatic tumor model, 2×10^6 Luc-CT26 or Luc-ID8 cells suspended in 200 μL of PBS were directly injected into the abdominal cavity of Balb/c mice or C57BL/6 mice. Tumor progression was assessed by intraperitoneal injection of luciferase substrate (1.5 mg mL^{-1}) and then recording the luminescence intensity using the IVIS Spectrum imaging system.

For *in vivo* peritoneal perfusion, tumor-bearing mice were first anesthetized with isoflurane according to the vendor's procedure. The infusion system catheter and the peritoneal perfusate, consisting of specific components in 100 mL of saline, were then preheated to 43 ºC. Subsequently, the inflow and outflow catheters were inserted into the left hypochondrium and the right iliac fossa of the abdominal wall, respectively. The perfusate was then infused using a roller pump at a rate of 3 mL/min for 30 minutes. After infusion, the inflow catheter was removed, and gentle pressure was applied to the abdomen to expel any excess fluid before the outflow catheter was withdrawn. Finally, the abdominal wall was closed using single sutures and disinfected with betadine [2].

In vivo cancer treatment

5 experimental groups of Luc-CT26 orthotopic transplantation tumor-bearing or Luc-CT26 abdominal metastatic tumor-bearing Balb/c mice (n=6) received the following treatments: group 1, no-treatment group (Control); group 2, intraperitoneal perfusion with TNIP (8 mM Ca^{2+}, 2 mM H_2O_2); group 3, HIP with 0.9% NaCl solution; group 4, HIP with 5-Fu (15 mg kg^{-1}); and group 5, HIP with TNIP (8 mM Ca^{2+}, 2 mM H_2O_2). The perfusion operation was kept at a fluid flow rate of 3 mL/min and 43 ºC for 30 min. The hyperthermic intraperitoneal perfusion with a standardized 30-minute perfusion duration as previously established. In the treatment of Luc-ID8

abdominal metastatic tumor model, cisplatin was used as the chemotherapeutic agents (3 mg kg^{-1}). The tumor progression and body weight of each mouse was monitored by using the IVIS Spectrum imaging system and electronic balance once every 4 days.

Analysis of anti-tumor immunity

For immune response analysis in post-treatment mice, a total of 25 abdominal metastatic tumor-bearing Balb/c mice were treated as abovementioned (n = 5). 7 days post-treatment, the mesenteric lymph nodes of these mice were collected for preparing single cell suspensions by following previously used procedures for subsequent immunofluorescence staining[3]. Ascites-derived cells were collected by peritoneal lavage with 5 mL 1×PBS, and the peritoneal lavage fluid was centrifuged at 300 g for 10 min at 4 ºC. Erythrocytes were lysed with commercial Ammonium-Chloride-Potassium (ACK) Lysing Buffe, and residual cells were resuspended with FACS buffer for immunofluorescence staining. The percentages of maturated DCs ($CD11C^+CD80^+CD86^+$), $CD3^+CD4^-CD8^+$ T cells, IFN-γ^+CD8^+ T cells ($CD3^+CD8^+$ IFN-γ^+), Tregs ($CD3^+CD4^+Foxp3^+$), M1 ($CD11b^+F4/80^+CD80^+$), M2 ($CD11b^+F4/80^+CD206^+$), GzB^+ NK cells ($CD45^+CD3^-CD49b^+GzB^+$) and MDSCs ($CD45^+CD11b^+Gr\text{-}1^+$) were analyzed by using the flow cytometry.

In vivo combined anti-PD-1 immunotherapy

Six groups of Luc-CT26 abdominal metastatic tumor-bearing Balb/c mice (n = 6) received the following treatments: group 1, untreated (Control); group 2, anti-PD-1 injection (anti-PD-1); group 3, HIP with 5-Fu; group 4, HIP with 5-Fu and anti-PD-1 injection (HIP+5-Fu+anti-PD-1); group 5, HIP treatment with TNIP (HIP+TNIP); group 6, HIP treatment with

TNIP and anti-PD-1 injection (HIP+TNIP+anti-PD-1). The dosages of 5-Fu and TNIP were the same as mentioned above, and anti-PD-1 was intravenously injected at a dose of 1 mg kg^{-1} at 1, 3, and 5 days after the HIP treatment. The tumor progression and body weight of each mouse was monitored by using the IVIS Spectrum imaging system and electronic balance once every 4 days. In the treatment of Luc-ID8 abdominal metastatic tumor model, cisplatin was used as the chemotherapeutic agents (3 mg kg^{-1}). The tumor progression and body weight of each mouse was monitored by using the IVIS Spectrum imaging system and electronic balance once every 4 days.

For evaluating the long-term immune memory effect induced by the combination treatment with HIP + TNIP + anti-PD-1, four cured mice were rechallenged with the same CT26 cells (2×10^6 cells) at 510 days after the primary treatments. Meanwhile, four healthy mice were subcutaneously injected with same CT26 cancer cells as the control group. The sizes of these rechallenged tumors were monitored as aforementioned. In addition, the peripheral blood of each mouse (~200 μL) was collected right before and 7 days post the inoculation of the second tumor for analyzing the percentage of memory T cells ($CD3^+CD8^+CD44^+CD62L^-$) via flow cytometry and the cytokines of TNF-α and IFN-γ via the ELISA assay.

TNIP-based HIP treatment in PBMCs supplemented PDOs

Advanced colorectal cancer samples and autologous PBMCs were collected at Shanxi provincial cancer hospital and approved by the local Ethics Committee. Written informed consent was obtained from patients. To establish PDOs, tumor samples were thoroughly minced, washed, and digested using Chongqing Kingmed tissue digestion solution, and collected via centrifugation. Then, the collected cells were resuspended in matrigel and seeded into multi-well

plates to generate organoids under the standard protocol. To investigate the anti-tumor efficacy of the TNIP-based HIP strategy in the PDOs model, established PDOs (50 μm in diameter) were received TNIP-HIP treatment (H_2O_2, 2 mM; Ca^{2+}, 8 mM; 43 °C, 1 h), and their supernatants were collected for LDH assay. After being cultures in fresh culture medium for another 24 h, these PDOs together untreated ones were further cultured with autologous PBMCs at an effector-target ratio of 4: 1. The morphology of PDOs in each subgroup was recorded via optical microscopy 1, 3, and 5 days after PBMCs addition. Then, the activation status of T cells in the added PBMCs were analyzed via flow cytometry, and their supernatants were collected for quantifying the secretion levels of IL-2, IFN-γ and TNF-α using respective ELISA kits.

Statistical analysis

All the data were presented as mean ± standard deviation (SD). One-way analysis of variance (ANOVA) was used to determine the significance of the difference. Statistical significance was set at $*p < 0.05$, $**p < 0.01$, $***p < 0.001$.

References

1. Xu L, Liu Y and Chen Z *et al.* Morphologically virus-like fullerenol nanoparticles act as the dual-functional nanoadjuvant for HIV-1 vaccine. *Adv Mater* 2013; **25**: 5928–36.
2. Hu X, Kang X and Zhao F *et al.* Heterogeneous cellular responses to hyperthermia support combined intraperitoneal hyperthermic immunotherapy for ovarian cancer mouse models. *Sci Transl Med* 2025; **17**: eadp2124.
3. He C, Duan X and Guo N *et al.* Core-shell nanoscale coordination polymers combine chemotherapy and photodynamic therapy to potentiate checkpoint blockade cancer immunotherapy. *Nat Commun* 2016; **7**: 12499.

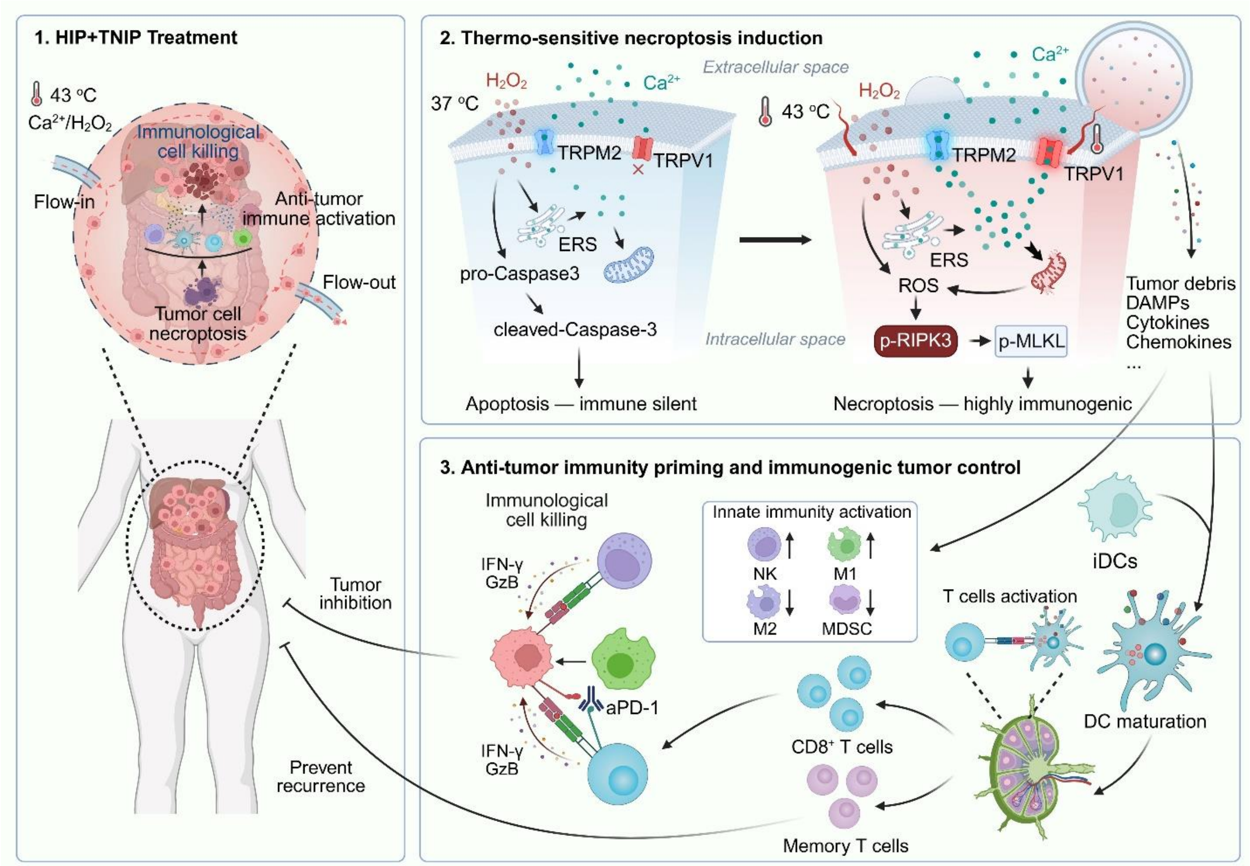

Figure S1. A scheme illustrating the mechanism of TNIP-based hyperthermic intraperitoneal immunotherapy. When heated to 43°C and perfused into the abdominal cavity (1), TNIP derived from a binary Ca^{2+}/H_2O_2 solution selectively induces necroptosis in cancer cells (2). This process generates highly immunogenic tumor debris and DAMPs, thereby eliciting robust adaptive antitumor immunity with enhanced DC maturation and $CD8^+$ CTL activation (3). Concurrently, TNIP-based HIP treatment activates robust innate immune responses characterized by TAM repolarization, downregulation of MDSCs, and NK cell activation. Consequently, it drives effective regression of diverse intraperitoneal tumors and induces long-term immune memory, particularly when combined with postoperative anti-PD-1 immunotherapy.

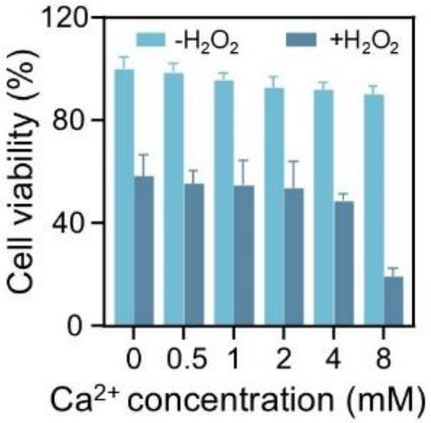

Figure S2. Cell viability of CT26 cells with indicated treatments. (n = 6)

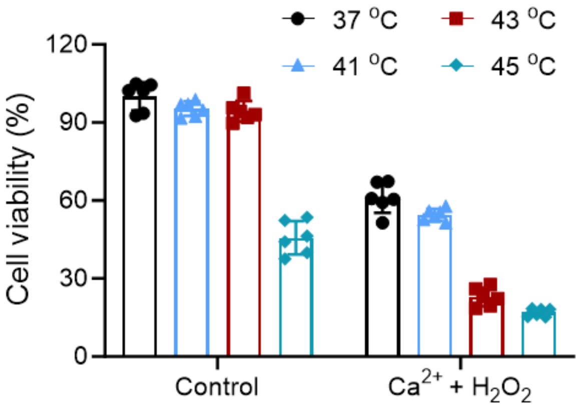

Figure S3. Relative cell viabilities of CT26 cells treated with Ca^{2+}/H_2O_2 co-incubation at indicated temperatures.

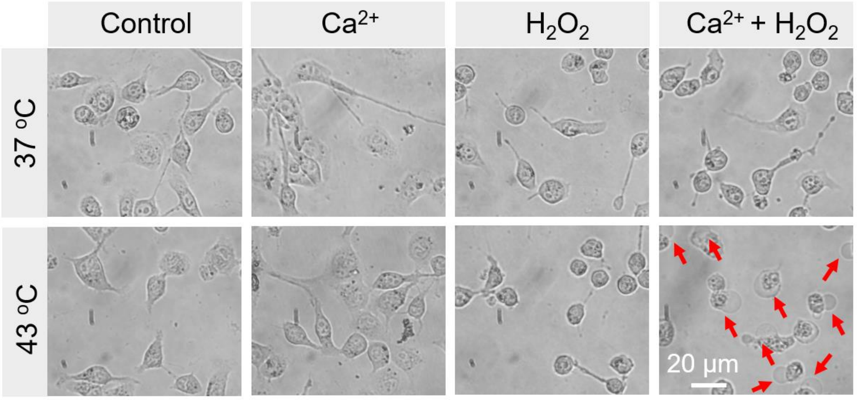

Figure S4. Representative optical images of CT26 cells with indicated treatments.

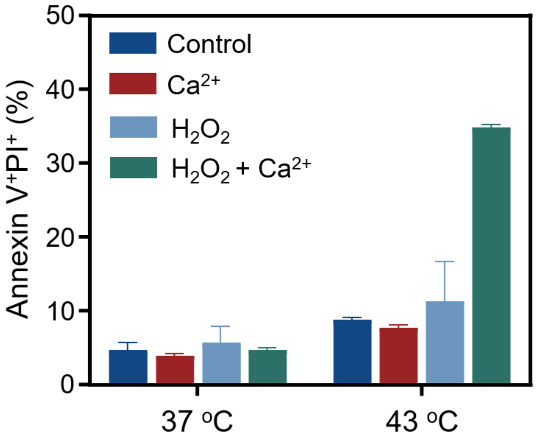

Figure S5. Flow cytometry analysis of proportions of Annexin V-FITC and PI dual-positive CT26 cells receiving indicated treatments. (n = 3)

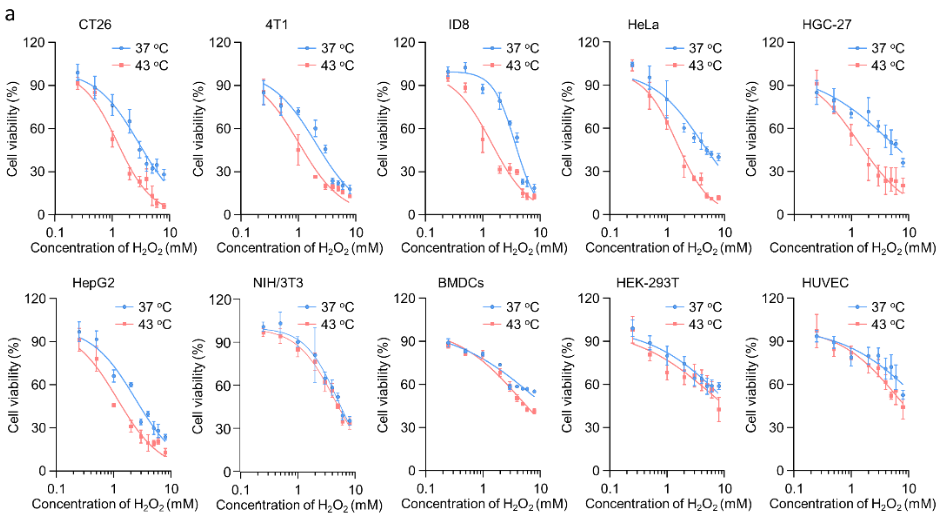

b

Cancer cells

Cell line	IC50 (mM) 37 °C	43 °C
CT26	2.90	1.24
4T1	2.05	1.04
ID8	3.70	1.45
HeLa	4.13	1.50
HGC-27	5.16	1.36
HepG2	2.40	1.17

Normal cells

Cell line	IC50 (mM) 37 °C	43 °C
NIH/3T3	4.96	4.42
BMDCs	>8	4.54
HEK-293T	>8	7.44
HUVEC	>8	6.83

Figure S6. (a) The cell viability of various normal cells and cancer cells with indicated treatments. (b) The IC50 values for various cancer cells and normal cells treated with gradient concentration of H_2O_2.

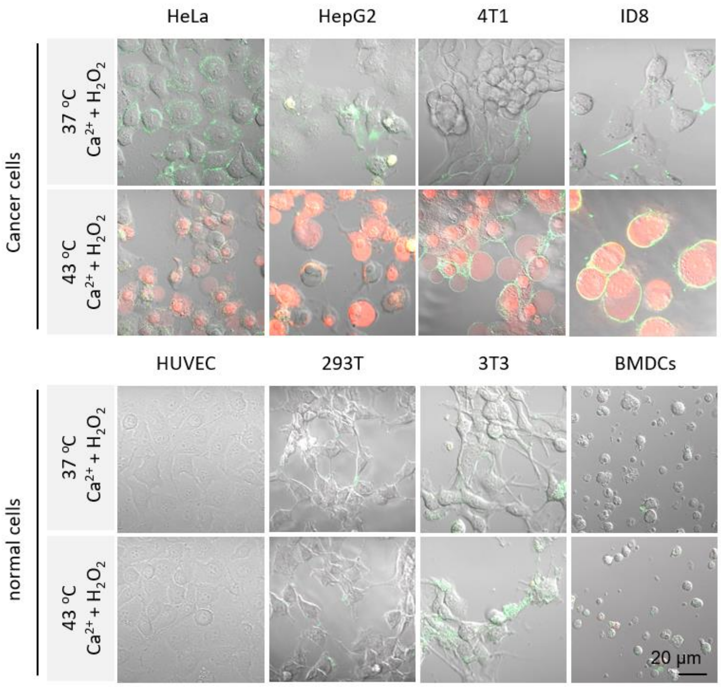

Figure S7. Representative confocal images of different cells stained by FITC-Annexin V (green) and PI (red) after Ca^{2+}/ H_2O_2 co-treatment at 37 ºC or 43 ºC.

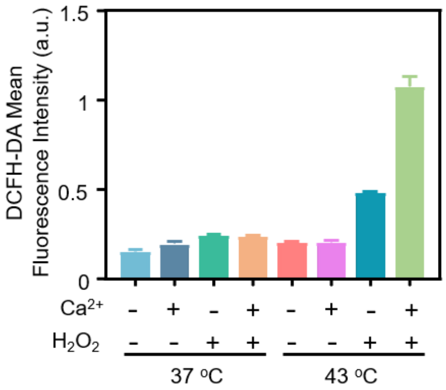

Figure S8. Mean fluorescence intensity (MFI) of intracellular ROS levels of CT26 cells with indicated treatments based on flow cytometry analysis. (n = 3)

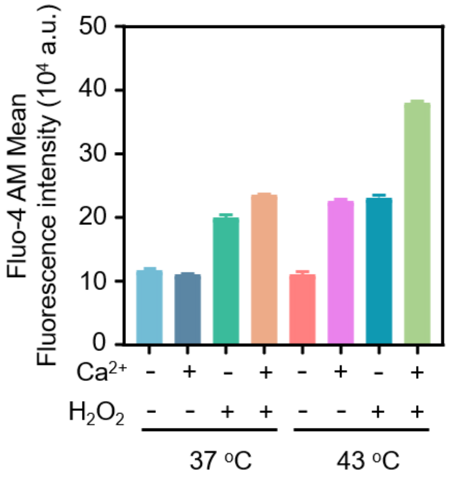

Figure S9. Flow cytometric analysis and corresponding quantification of the mean fluorescence intensity (MFI) of intracellular Ca^{2+} levels in CT26 cells post indicated treatments. (n = 3)

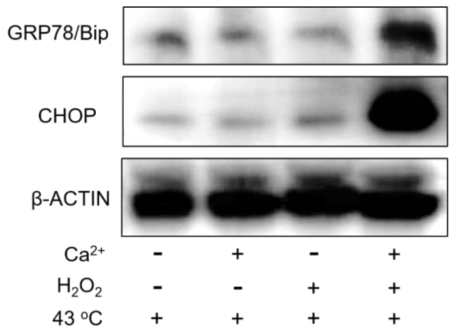

Figure S10. Western blotting of CHOP and GRP78/Bip expression levels in CT26 cells with indicated treatments.

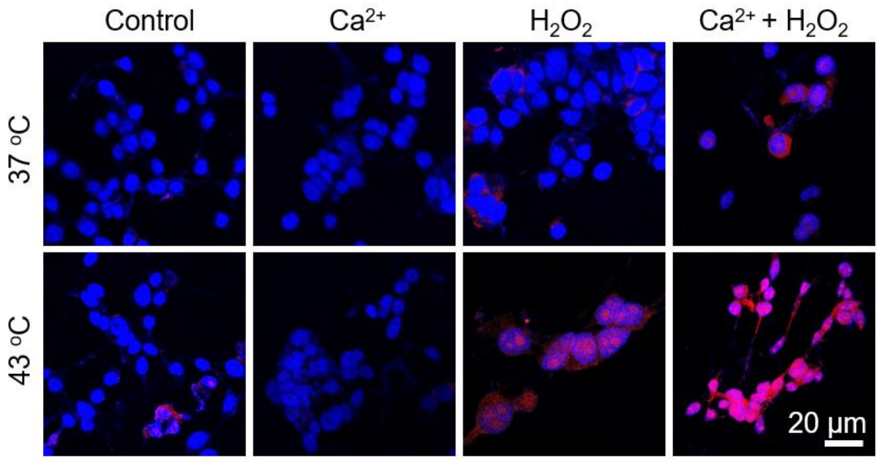

Figure S11. Confocal images of CT26 cells stained by MitoSOX Red (red) and DAPI (blue) after various treatments.

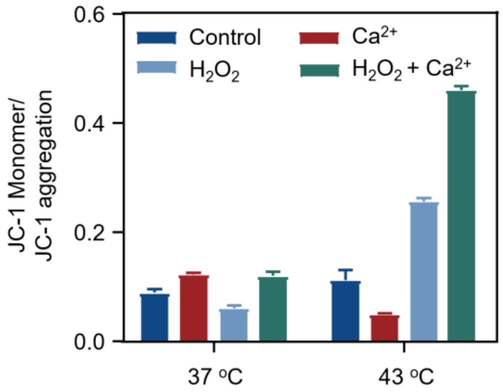

Figure S12. Flow cytometry analysis of JC-1 stained CT26 cells post indicated treatments.

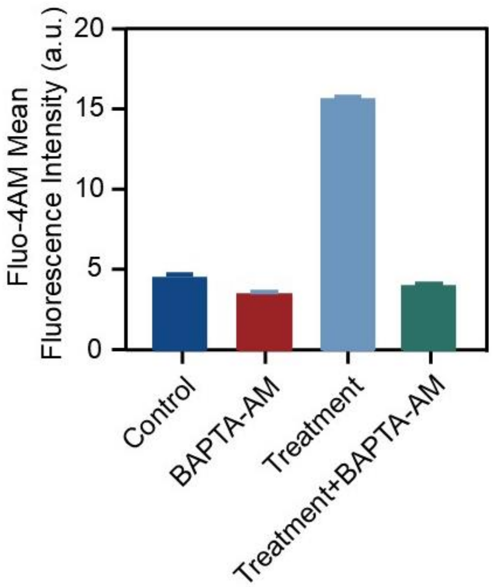

Figure S13. Flow cytometric analysis and corresponding quantification of the mean fluorescence intensity (MFI) of intracellular Ca^{2+} levels in CT26 cells post indicated treatments. (n = 3)

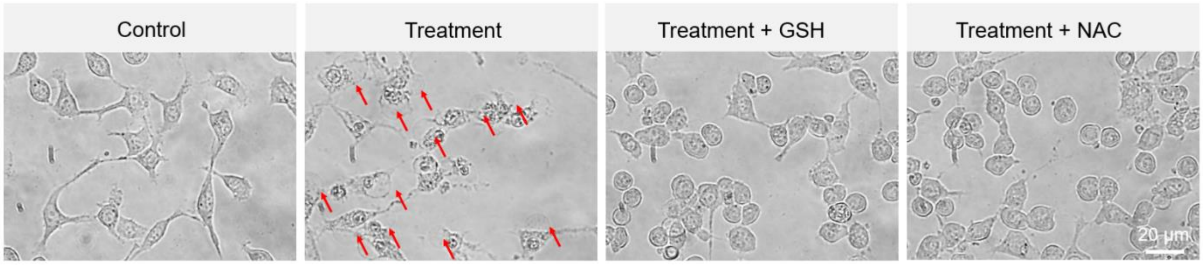

Figure S14. Representative optical images of CT26 cells treated with H_2O_2/Ca^{2+} co-incubation at 43 °C in the presence and absence of GSH or NAC pretreatments.

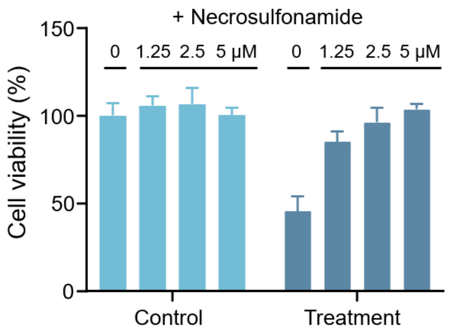

Figure S15. Relative cell viabilities of CT26 cells treated with Ca^{2+}/H_2O_2 co-incubation at 43 ºC in the presence and absence of necrosulfonamide pretreatment. (n = 6)

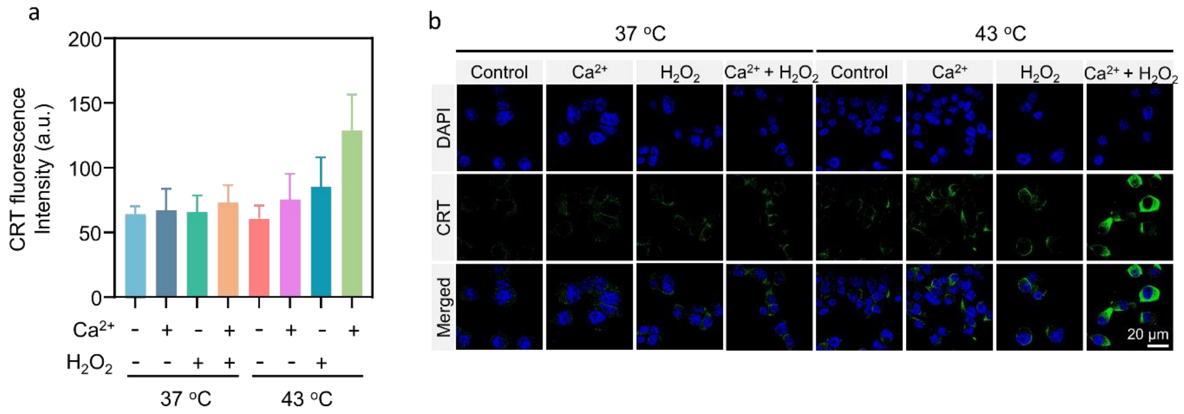

Figure S16. Relative fluorescence intensity (a) and confocal imaging (b) of cellular CRT expression levels in CT26 cells post indicated treatments.

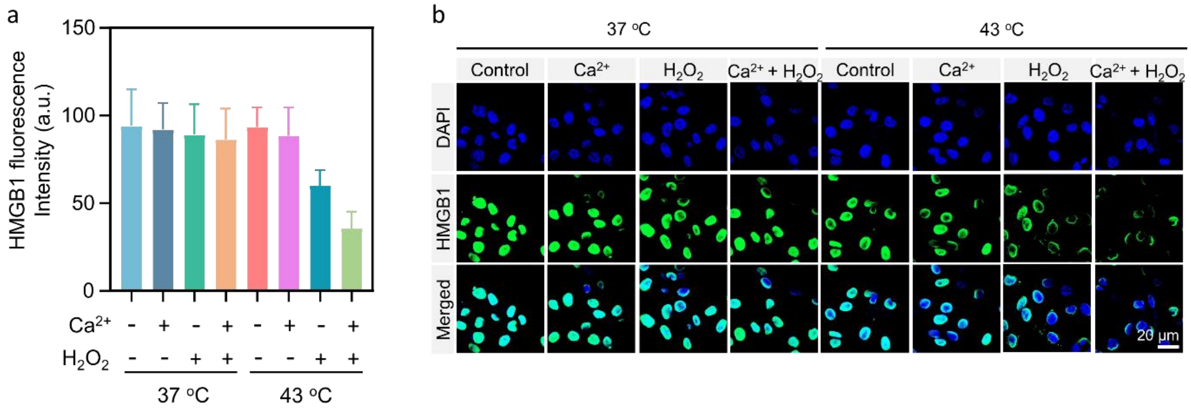

Figure S17. Relative fluorescence intensity (a) and confocal imaging (b) of intranuclear HMGB1 levels in CT26 cells post indicated treatments.

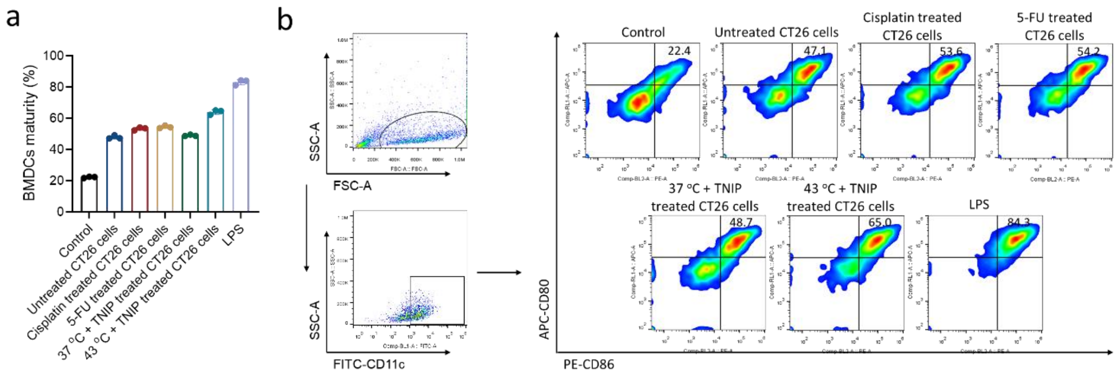

Figure S18. Corresponding quantification results (a) and representative flow cytometric plots (b) of CD11c$^+$CD86$^+$CD80$^+$ DC cells population in BMDCs with indicated treatments.

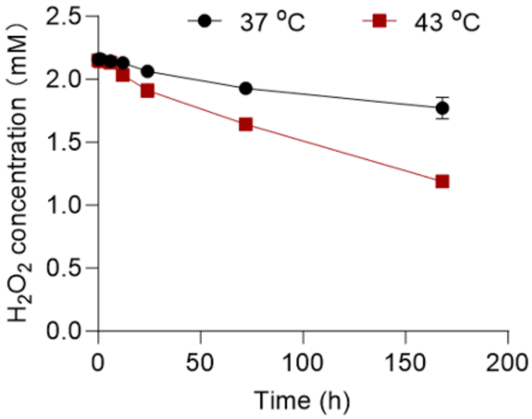

Figure S19. Content variation curve of H_2O_2 when incubated at 37 or 43 °C.

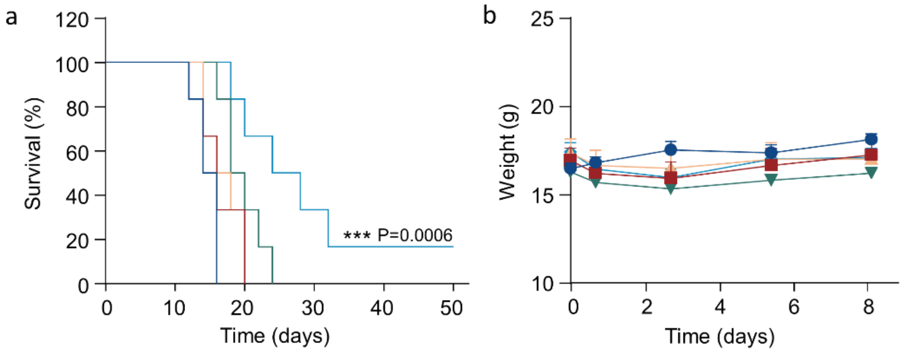

Figure S20. Survival rate (a) and body weights (b) of mice receiving the indicated treatments.

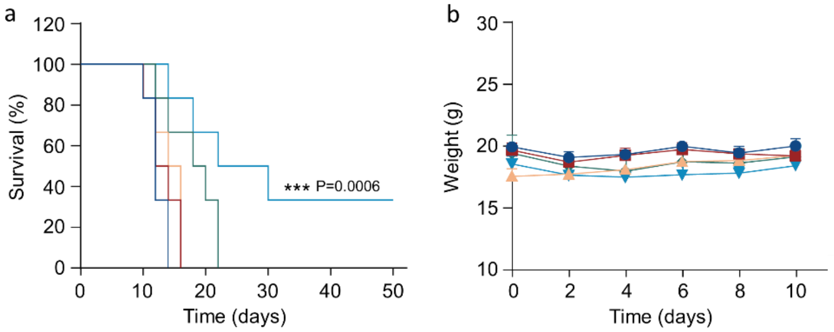

Figure S21. Survival rate (a) and body weights (b) of mice receiving the indicated treatments.

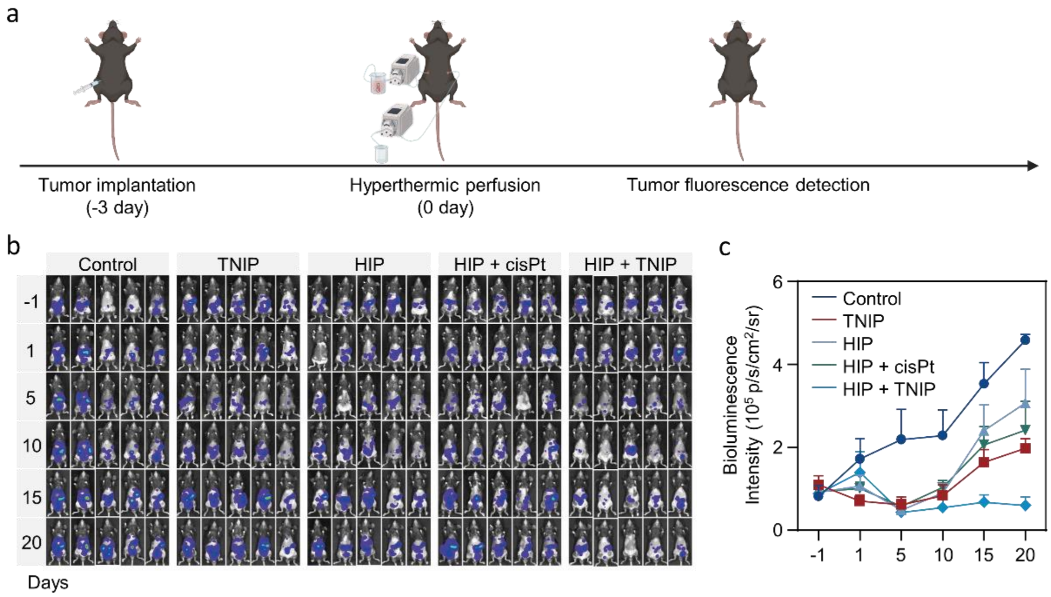

Figure S22. *In vivo* TNIP-based HIP treatment in Luc-ID8 abdominal metastatic tumor model (n = 5). (a) Schematic illustration of the experimental schedule for the treatment in Luc-ID8 abdominal metastatic tumor model. (b) Bioluminescence imaging of mice post indicated treatments. (c) Semi-quantitative analysis of bioluminescence intensity of mice post indicated treatments based on the treatments showing in Figure S13B.

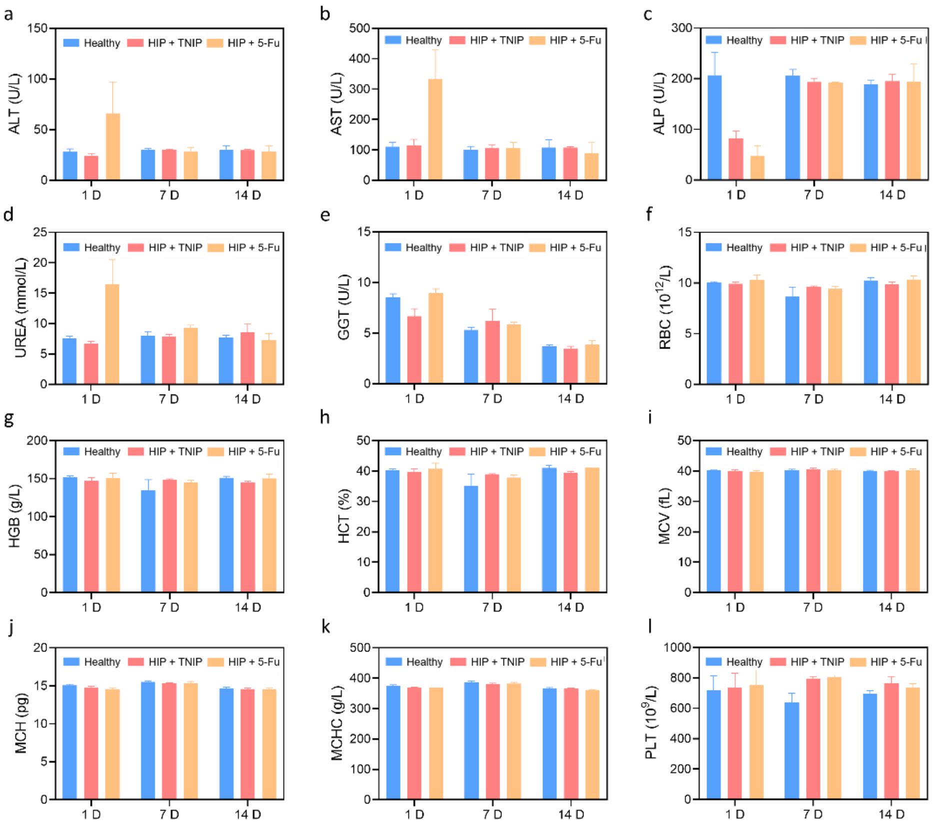

Figure S23. Blood biochemistry analysis and complete blood panel analysis of healthy and TNIP-HIP treated mice. (a) alanine aminotransferase (ALT), (b) aspartate transaminase (AST), (x) alkaline phosphatase (ALP), (d) urea, (e) γ-glutamyl transferase (GGT), (f) red blood cells (RBC), (g) hemoglobin content (HBG), (h) hematocrit (HCT), (i) mean corpuscular volume (MCV), (j) mean corpuscular hemoglobin (MCH), (k) mean corpuscular hemoglobin concentration (MCHC), (l) platelets (PLT). (n = 3)

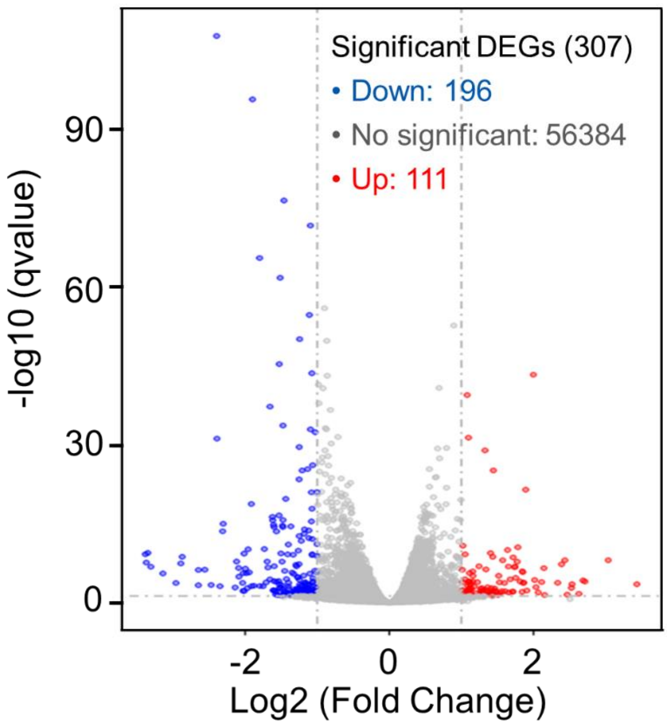

Figure S24. A volcano plot showing the overall distribution of DEGs in the mice with TNIP-based HIP treatment compared to that of control mice.

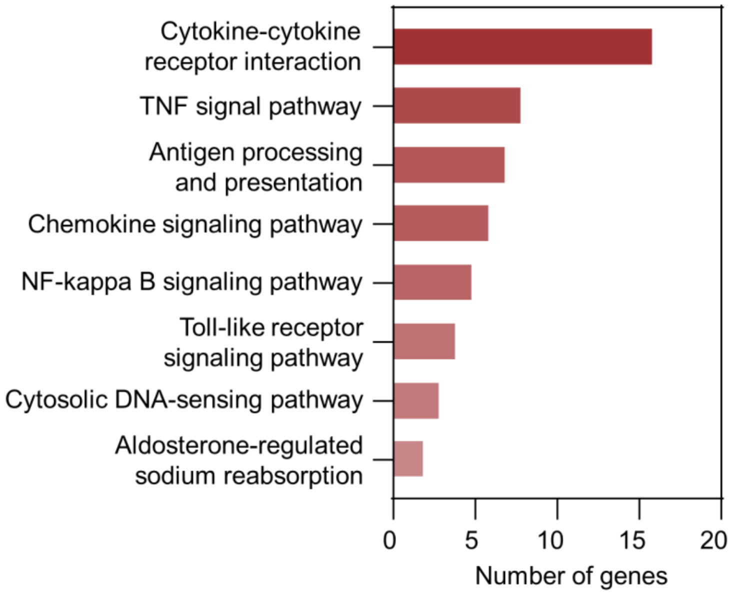

Figure S25. KEGG enrichment analysis of the DEGs in mice with TNIP-based HIP treatment and control treatment.

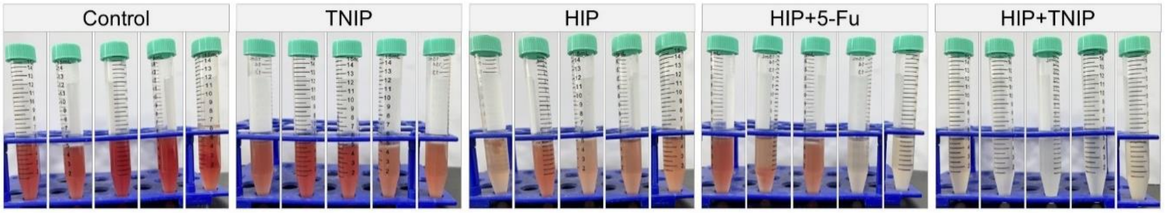

Figure S26. Optical images of peritoneal lavage fluids of mice receiving indicated treatments.

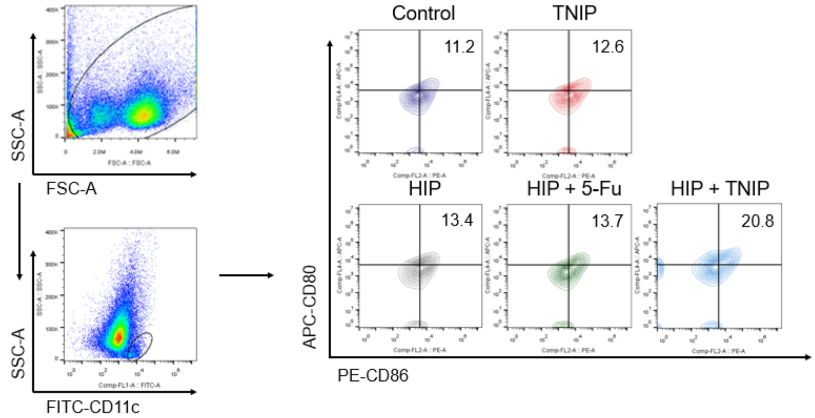

Figure S27. Flow cytometry gating strategy and representative flow cytometric plots of DCs maturation status in the mesenteric lymph nodes of mice post indicated treatments.

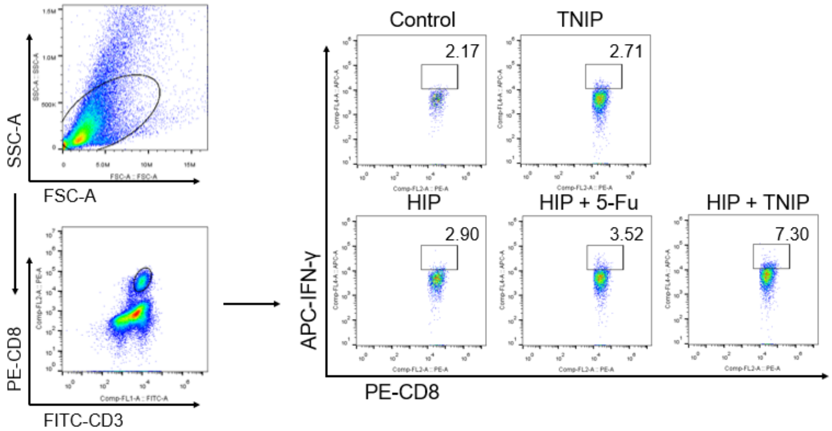

Figure S28. Flow cytometry gating strategy and representative flow cytometric plots of $CD3^+CD8^+IFN\text{-}\gamma^+$ T cells in peritoneal lavage fluids of mice after various treatments as indicated.

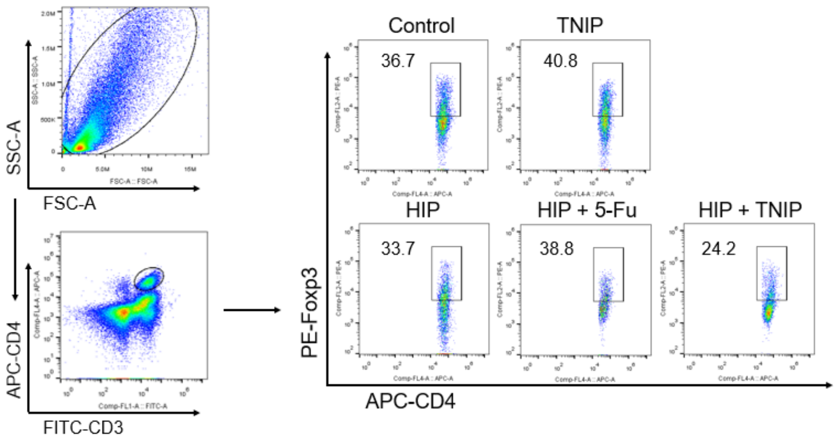

Figure S29. Flow cytometry gating strategy and representative flow cytometric plots of $CD3^+CD4^+Foxp3^+$ Tregs in peritoneal lavage fluids of mice after various treatments as indicated.

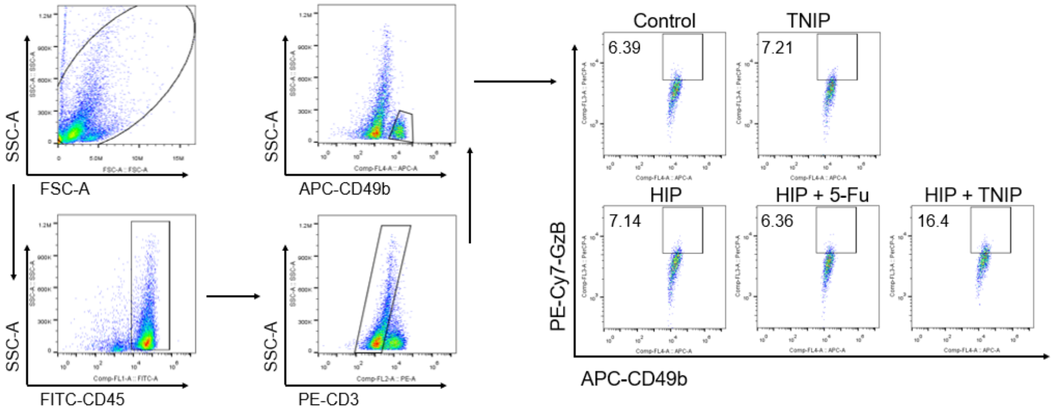

Figure S30. Flow cytometry gating strategy and representative flow cytometric plots of $CD45^+CD3^-CD49b^+GzB^+$ NK cells in peritoneal lavage fluids of mice after various treatments as indicated.

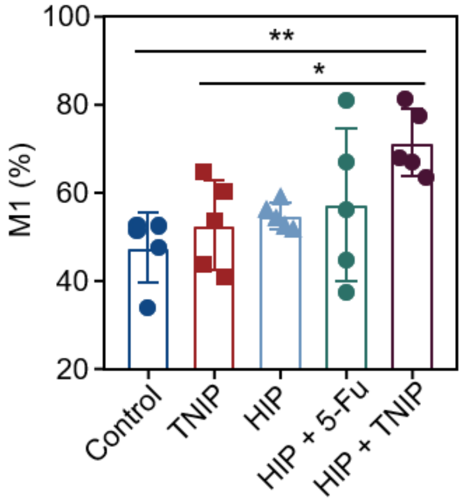

Figure S31. The frequencies of M1 macrophages in peritoneal lavage fluids of mice after various treatments as indicated. (n = 5)

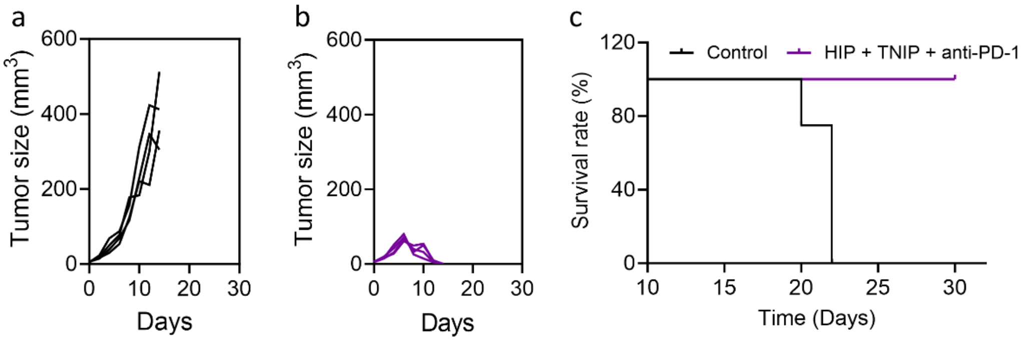

Figure S32. (a and b) The individual tumor growth curves of rechallenged CT26 tumor in healthy control mice and mice cured by the HIP+TNIP+aPD-1 treatment. (c) Survival rate of mice receiving indicated treatments.

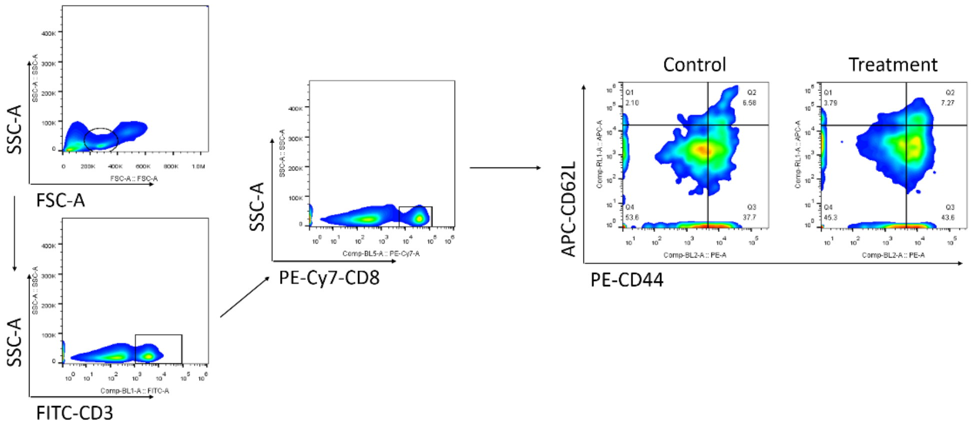

Figure S33. Flow cytometry gating strategy and representative flow cytometric plots of $CD3^{+}CD8^{+}CD44^{+}CD62L^{-}$ T_{EM} cells in peripheral blood of mice at the day right before rechallenging mice with CT26 cancer cells.

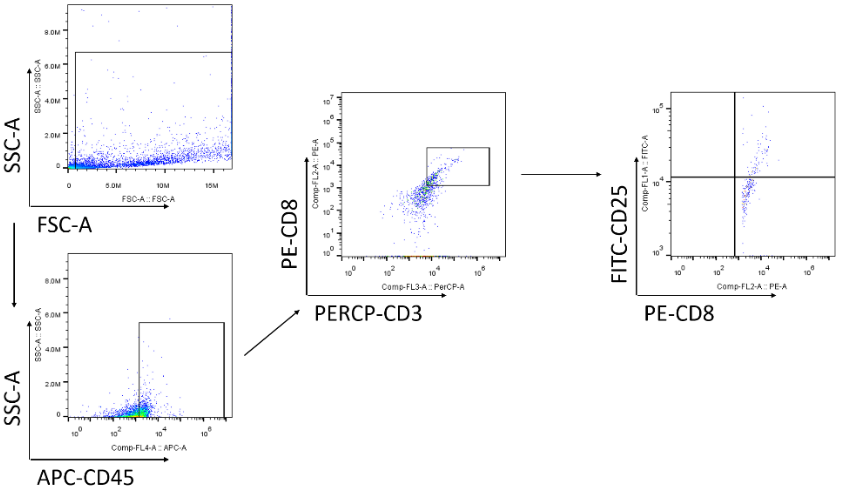

Figure S34. Flow cytometry gating strategy of CD45$^+$CD3$^+$CD8$^+$CD25$^+$ T cells population in PBMCs.

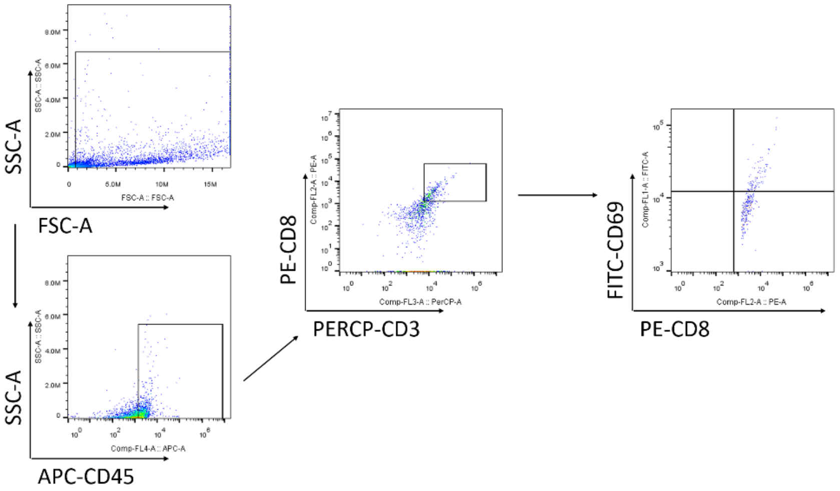

Figure S35. Flow cytometry gating strategy of CD45$^+$CD3$^+$CD8$^+$CD69$^+$ T cells population in PBMCs.
